# Krüppel-like factor 15 integrated autophagy and gluconeogenesis to maintain glucose homeostasis under 20-hydroxyecdysone regulation

**DOI:** 10.1371/journal.pgen.1010229

**Published:** 2022-06-13

**Authors:** Xiao-Pei Wang, Zhen Huang, Yan-Li Li, Ke-Yan Jin, Du-Juan Dong, Jin-Xing Wang, Xiao-Fan Zhao

**Affiliations:** Shandong Provincial Key Laboratory of Animal Cells and Developmental Biology, School of Life Sciences, Shandong University, Qingdao, China; University of Kentucky, UNITED STATES

## Abstract

The regulation of glycometabolism homeostasis is vital to maintain health and development of animal and humans; however, the molecular mechanisms by which organisms regulate the glucose metabolism homeostasis from a feeding state switching to a non-feeding state are not fully understood. Using the holometabolous lepidopteran insect *Helicoverpa armigera*, cotton bollworm, as a model, we revealed that the steroid hormone 20-hydroxyecdysone (20E) upregulated the expression of transcription factor Krüppel-like factor (identified as *Klf15*) to promote macroautophagy/autophagy, apoptosis and gluconeogenesis during metamorphosis. 20E via its nuclear receptor EcR upregulated *Klf15* transcription in the fat body during metamorphosis. Knockdown of *Klf15* using RNA interference delayed pupation and repressed autophagy and apoptosis of larval fat body during metamorphosis. KLF15 promoted autophagic flux and transiting to apoptosis. KLF15 bound to the KLF binding site (KLF bs) in the promoter of *Atg8* (autophagy-related gene 8/LC3) to upregulate *Atg8* expression. Knockdown *Atg8* reduced free fatty acids (FFAs), glycerol, free amino acids (FAAs) and glucose levels. However, knockdown of *Klf15* accumulated FFAs, glycerol, and FAAs. Glycolysis was switched to gluconeogenesis, trehalose and glycogen synthesis were changed to degradation during metamorphosis, which were accompanied by the variation of the related genes expression. KLF15 upregulated phosphoenolpyruvate carboxykinase (*Pepck*) expression by binding to KLF bs in the *Pepck* promoter for gluconeogenesis, which utilised FFAs, glycerol, and FAAs directly or indirectly to increase glucose in the hemolymph. Taken together, 20E via KLF15 integrated autophagy and gluconeogenesis by promoting autophagy-related and gluconeogenesis-related genes expression.

## Author summary

Glucose is the direct substrate for energy production in animal and humans. Autophagy and gluconeogenesis are known to help organisms maintaining energy substrates; however, the mechanism of integration of autophagy and gluconeogenesis is unclear. Holometabolous insects stop feeding during metamorphosis under steroid hormone 20-hydroxyecdysone (20E) regulation, providing a good model for the study. Using lepidopteran insect *Helicoverpa armigera*, cotton bollworm, as a model, we revealed that Krüppel-like factor 15 (KLF15) integrated autophagy and gluconeogenesis to maintain glucose homeostasis under 20E regulation. 20E increased *Klf15* expression, and KLF15 in turn promoted autophagy-related and gluconeogenesis-related genes expression during metamorphosis. Autophagy and apoptosis of the fat body provided substrates for gluconeogenesis. This work clarified the important functions and mechanisms of KLF15 in autophagy and glycometabolism reprogramming for glucose homeostasis after feeding stop during insect metamorphosis.

## Introduction

Glucose is an essential energy substance in animals and humans, which directly or indirectly produces energy for metabolic processes. Some tissues, such as the brain and red blood cells in mammals, rely on glucose to meet their energy requirements. Glycometabolism homeostasis maintains the blood glucose levels. Glucose is digested from food in normal feeding conditions and can be used directly *in vivo* through glycolysis to produce pyruvic acid (PA) and adenosine triphosphate (ATP). The circulating glucose is also produced from glycogenolysis under the catalysis of glycogen phosphorylase (GP) [1], and gluconeogenesis, the process of producing glucose from non-carbohydrate precursors, such as lactic acid (LA), glycerol, PA and free amino acids (FAAs) [2, 3]. Glycogen storage is depleted in 10 h during fasting [4], and gluconeogenesis becomes the predominant and almost exclusive glucose production process during fasting. Consequently, glycometabolism must be reprogrammed during fasting. Autophagy is also the important mechanism for supplying energy substrates during fasting and nutrients insufficient, however, the integration of autophagy and gluconeogenesis is unclear.

Krüppel-like factors (KLFs) are transcriptional regulators with three C2H2 zinc finger motifs at their carboxyl terminus, allowing them to bind to GC-rich DNA with a consensus binding site of CACCC (KLF bs) [5, 6]. KLFs respond to nuclear receptors of steroid and thyroid hormones to promote or repress downstream genes transcription [7]. KLFs play roles to extend lifespan in an autophagy-dependent manner in *Caenorhabditis elegans* [8]. KLFs actively play a role in the metabolic regulation. KLF 3 is essential for fat metabolism in *C*. *elegans* [9]. In mice, KLF15 is an important transcription factor involved in glycometabolism, especially in the regulation of gluconeogenesis [10]. The hepatic abundance of KLF15 increases during fasting and promotes the transcription of genes encoding the key enzymes of gluconeogenesis, i.e., glucose-6-phosphatase (*G6p*) and phosphoenolpyruvate carboxykinase (*Pepck*), to strengthen the occurrence of gluconeogenesis [11]. KLF15 also increases the expression of genes encoding amino acids catabolic enzymes in the liver to provide abundant substrates for gluconeogenesis [12, 13]. KLF15 suppresses cell growth in lung adenocarcinoma [14]. However, the role and mechanism of KLF15 regulating autophagy are unclear.

Insect metamorphic development presents good model for the study of autophagy and gluconeogenesis. When holometabolous insects stop feeding and transfer from larvae to adults, the energy for larvae survival and adult tissue development is derived from stored materials. Larval tissues are degraded by programmed cell death (PCD), including autophagy [15] and apoptosis [16]. As the homologous tissue of mammalian liver, the insect fat body is the primary tissue for sensing various hormones, nutrient storage [17], and glycometabolism [18]. Lipid droplets (LD) are the primary lipid storage organelles in the fat body cells, and triglycerides are the primary lipid storage components [19]. The insect molting hormone, steroid hormone 20-hydroxyecdysone (20E), is the principal regulator of metamorphosis [20, 21]. The 20E titer continues to rise during larvae-pupae transition [22]. 20E binds its nuclear receptor (EcR) and form a transcription complex with ultraspiracle isoform 1 (USP1) to bind with ecdysone response element (EcRE) [23] to initiate downstream genes expression that promotes metamorphosis [24]. High concentrations of 20E promotes the transformation of tissues from autophagy to apoptosis [25]. Glucose levels in the hemolymph increase after feeding stop during the larva-to-adult transition [26], and larval tissues are degraded by autophagy and apoptosis. Despite extensive research in 20E’s regulatory roles in metamorphosis, the mechanism by which 20E regulates gluconeogenesis and its relationship with larval fat body PCD, including autophagy and apoptosis remain unknown.

In the present study, we used the holometabolous insect, *Helicoverpa armigera*, cotton bollworm, as a model to reveal the mechanisms of glycometabolism reprogramming and fat body PCD under 20E regulation. We revealed that 20E, via KLF15, promoted larval fat body degradation by autophagy and apoptosis to produce substrates for gluconeogenesis during insect metamorphosis.

## Results

### Screen of KLFs involved in gluconeogenesis

By BLAST (Basic Local Alignment Search Tool), we screened five KLFs in the *H*. *armigera* genome using human KLFs as a guide, which are named as Krüppel-like factor luna (*Luna*), Dendritic arbor reduction protein (*Dar*), *Klf8*, *Klf9* and *Klf16*. KLF9 was renamed as KLF15 because it was not clustered with *Homo sapiens* KLF9 (S1 Fig), but was clustered with *Drosophila melanogaster* KLF15 and has sequence similarity with *D*. *melanogaster* and *H*. *sapiens* KLF15 (S2 Fig). *Klf15* expression was higher in the fat body at the sixth instar 96 h (6th-96 h) compared to 6th-24 h, the expression of *Luna* and *Klf16* in the fat body were also significantly higher at the 6th-96 h (S3A–S3E Fig). To identify KLF involved in gluconeogenesis, we knocked down *Luna*, *Klf15*, and *Klf16* and measured glucose levels in the hemolymph (S3F Fig). Glucose levels in the hemolymph were significantly reduced after knocked down *Klf15* and *Klf16* (S3G Fig), and *dsKlf15* significantly decreased the expression of *Pepck* in the fat body, whereas *dsKlf16* did not (S3H Fig). Thus, KLF15 was chosen for our further study in this work.

### *Klf15* was highly expressed in the fat body during metamorphosis under 20E regulation

**Fig 1:**
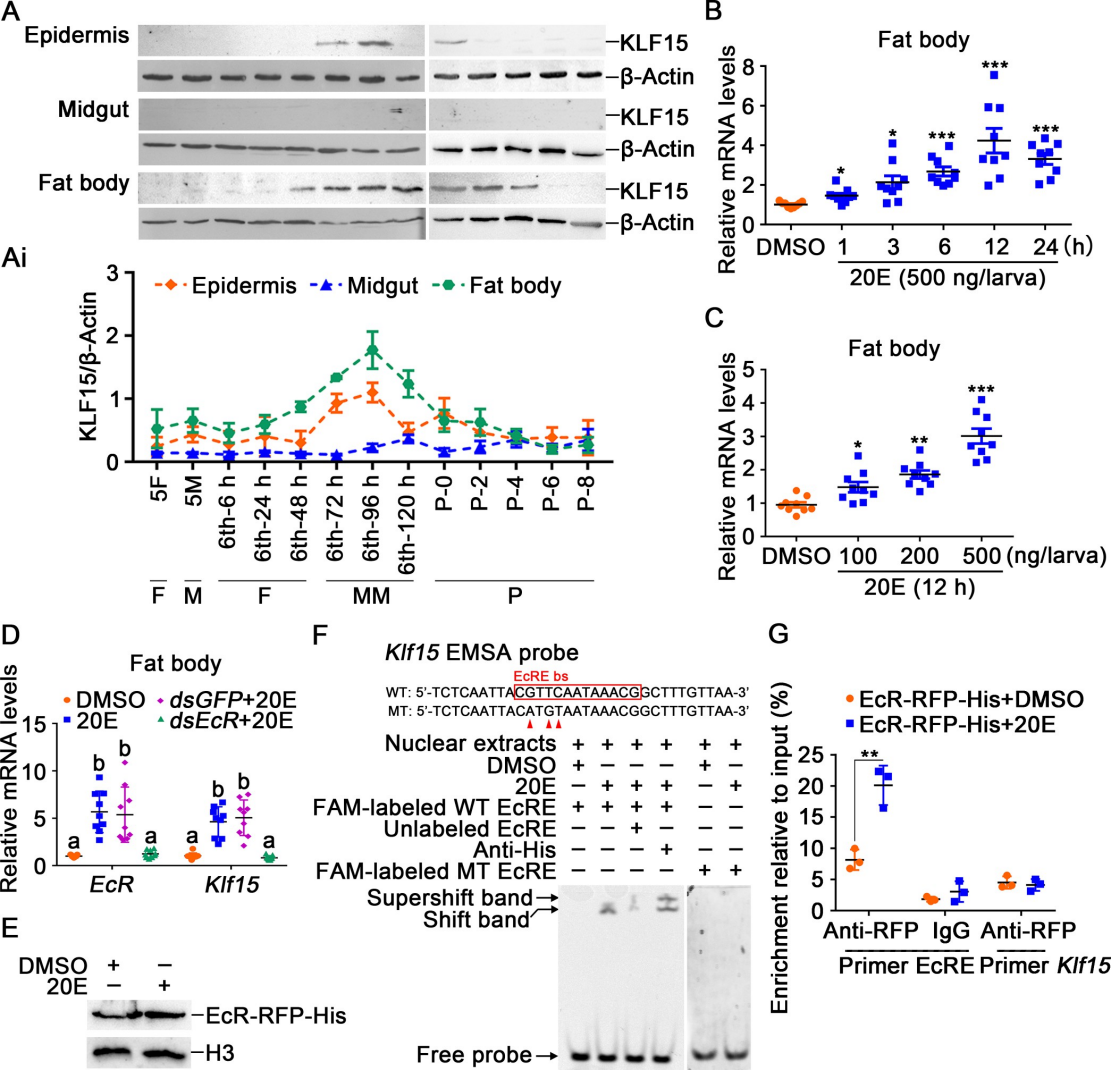
20E upregulated the expression of *Klf15*. **A.** The protein profiles of KLF15 in the epidermis, midgut, and fat body detected using western blotting after 12.5% SDS-PAGE. 5F: fifth instar feeding larvae; 5M: fifth instar molting larvae; 6th-6 h to 120 h: sixth instar larvae at different stages; P0 to P8: 0 to 8-day-old pupae. F: feeding; M: molting; MM: metamorphic molting; P: pupae. **Ai.** Quantification of KLF15 in A using Image J software. **B.** Time course of the *Klf15* expression in the fat body after 20E (500 ng/larva) induction. DMSO was used as the control. **C.** The expression of *Klf15* in the fat body under stimulation with different concentrations of 20E for 12 h. **D.** Knockdown of *EcR* in the fat body by *dsEcR* (3 μg/larva) followed by stimulation with 20E (500 ng/larva) for 12 h to detect the expression of *Klf15*. **E.** Nuclear proteins from EcR-RFP-His overexpressed cells were extracted for EMSA. **F.** EcRE on the *Klf15* promoter bound to EcR detected by EMSA assay. WT and MT represent EcRE probe and EcRE mutant probe, respectively. **G.** ChIP assay showing 20E promoted *Klf15* expression via EcR binding to EcRE and detected by qRT-PCR. Primer EcRE is the sequence containing EcRE. Primer *Klf15*, as non-EcRE control targeting to *Klf15* open reading frame (ORF).

The expression profiles of KLF15 during metamorphosis was examined to analyze its function in development. KLF15 levels increased in the epidermis and fat body during metamorphosis (Fig 1A and 1Ai), indicating that KLF15 plays an important role during metamorphosis. To understand the regulation of 20E on KLF15, 20E was injected into the larval hemocoel. *Klf15* expression was increased in a time and 20E-concentration dependent manner (Fig 1B and 1C), indicating that 20E promotes *Klf15* expression. To confirm 20E upregulating *Klf15* expression, we knocked down 20E nuclear receptor *EcR* in the fat body. Results showed that the expression of *Klf15* was decreased after knockdown of *EcR* (Fig 1D). Four EcRE that could be combined with EcR in the *Klf15* promoter were further predicted by reported EcRE consensus sequences and JASPAR transcription factor database [27, 28] (S4 Fig). Following successfully expressing EcR-RFP-His in *H*. *armigera* epidermal cell line (HaEpi) (Fig 1E), we found that the EcRE with the sequence 5’-CGTTCAATAAACG-3’ was able to be combined by the overexpressed EcR-RFP-His in the nuclear extracts after stimulation with 20E using EMSA, and the antibody against His tag produced a supper shift band. However, after point mutations were introduced into the EcRE motif according to the method [29, 30], whether 20E or dimethyl sulfoxide (DMSO) was added, the probe was not able to be combined by nuclear proteins (Fig 1F). A chromatin immunoprecipitation (ChIP) assay showed that EcR-RFP-His bound more EcRE in the *Klf15* promoter under 20E treatment than it did in the DMSO treatment control (Fig 1G), which confirmed that 20E upregulated *Klf15* expression through its nuclear receptor EcR.

### KLF15 promoted pupation and fat body autophagy and apoptosis

**Fig 2:**
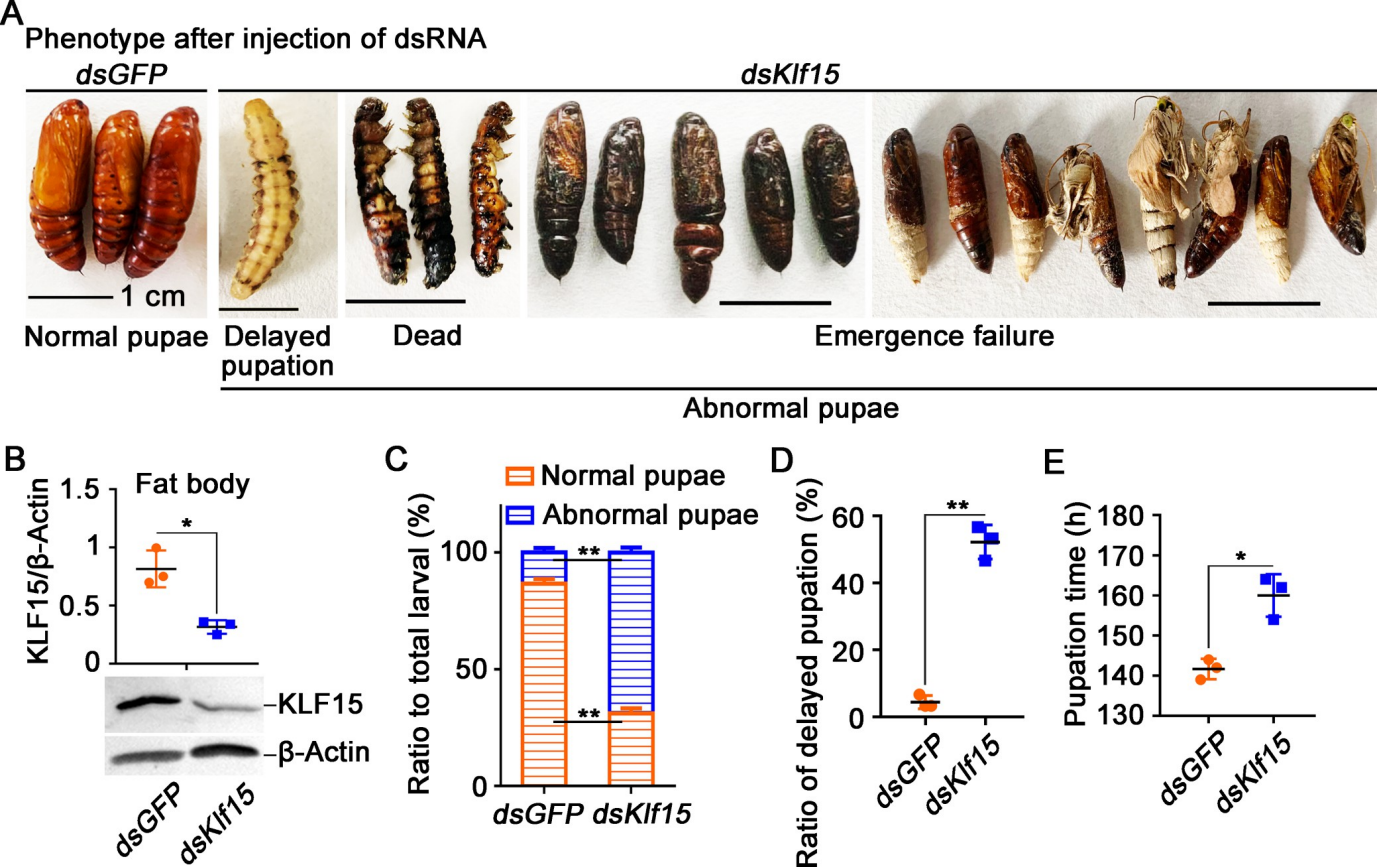
Knockdown of *Klf15* delayed pupation. **A.** Phenotypes after injection of *Klf15* dsRNA from 6th-6 h larva to 72 h; the ruler represents 1 cm, *dsGFP* was used as the control. **B.** Western blotting validation of the interference efficiency in the fat body after the third dsRNA injection. **C.** Ratio of phenotypes, abnormal pupae include larvae that die during the prepupa stage, dead pupae and chimeric pupae that cannot normally emerge. **D.** The ratio of delayed pupation after the dsRNA injection. **E.** The time at which the larvae pupated after knockdown *GFP* and *Klf15*. The data were obtained in triplicate with thirty larvae each time.

To determine the role of KLF15 in metamorphosis, we knocked down *Klf15* by injecting *dsKlf15* into the 6th-6 h larval hemocoel. The larvae showed delayed pupation and metamorphic failure after knockdown of *Klf15* (Fig 2A). *Klf15* was confirmed as being knocked down significantly in the fat body according to its protein levels (Fig 2B). After *dsKlf15* injection, 68.9% larvae had abnormal pupation, including death in the pre-pupal stage and pupal stage, as well as failure to eclosion normally (Fig 2C), 52.2% larvae showed a significant delay in pupation for an average of 20 h, and then dead at pupal or adult stages (Fig 2D and 2E).

**Fig 3:**
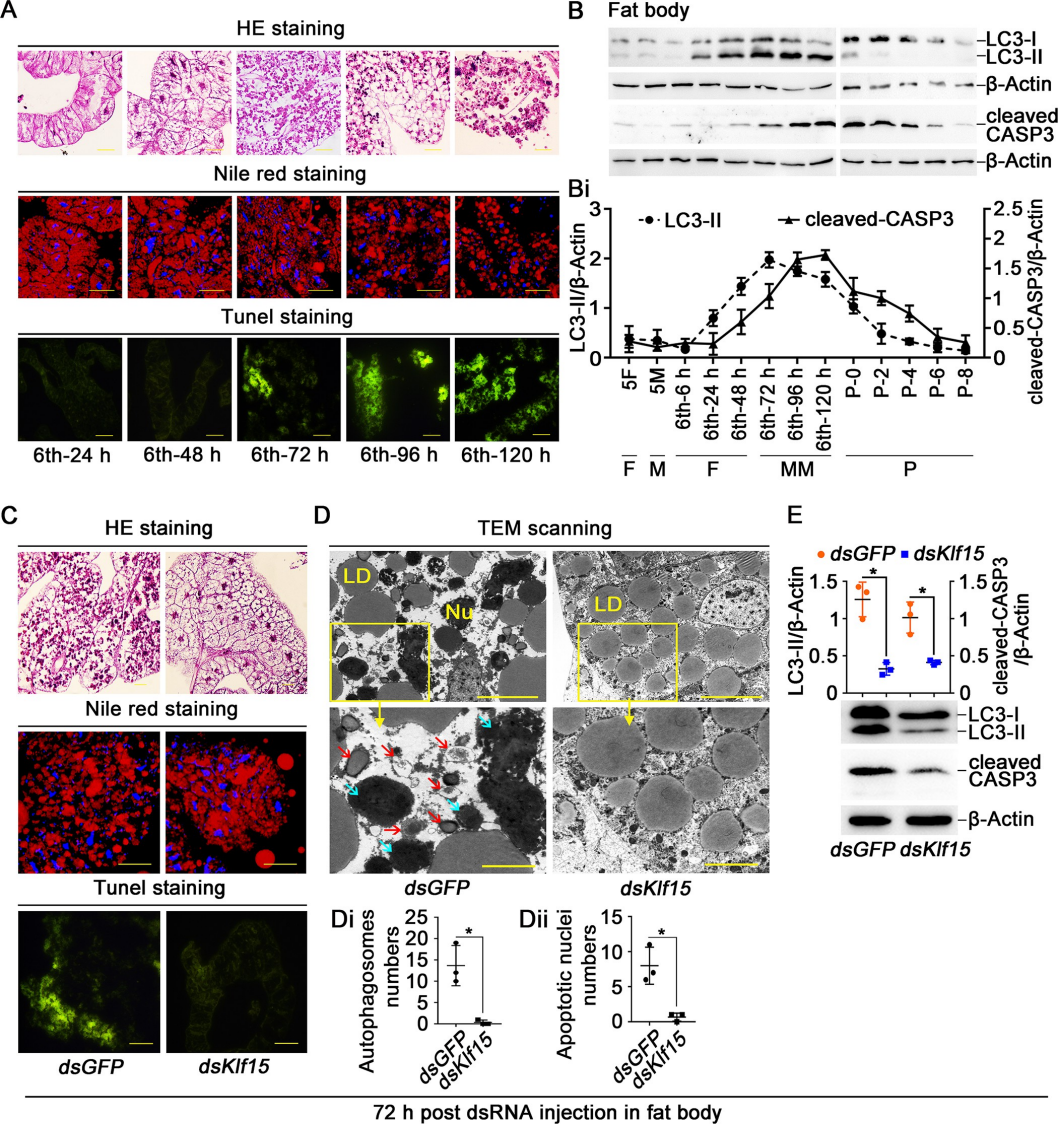
Knockdown of *Klf15* repressed fat body degradation. A. The morphological changes of fat body were assessed by HE staining, Nile red staining and TUNEL staining, the ruler represents 50 μm in the HE staining. Nile red stained for intracellular LD, the ruler represents 50 μm. The apoptotic cells in the fat body were determined by TUNEL staining assay, the ruler represents 100 μm. **B.** Western blotting detection of LC3-II and cleaved-CASP3 protein levels in the fat body using anti-LC3 and anti-CASP3 antibodies, with β-Actin as protein control after 15% SDS-PAGE. **Bi.** Quantification of the data in B. **C.** HE staining, Nile red staining and TUNEL staining showing fat body morphology after knockdown *Klf15*, observed after first injection dsRNA for 72 h. **D.** TEM observation after injection with *dsKlf15* in the fat body. The bars in the wide field view represented 100 μm, in the small field view represented 40 μm. The red arrows indicated autophagosomes, the blue arrow represented the apoptotic nuclei. Nu: nucleus, LD: lipid droplets. **Di.** Counted the autophagosomes contained in three different sets of images. The area of each image is about 0.09 mm^2^. **Dii.** Counted the apoptotic nuclei of *dsGFP* and *dsKlf15*. **E.** After knockdown *dsGFP* and *dsKlf15*, western blotting detected LC3-II and cleaved-CASP3. 72 h post dsRNA injection in fat body: 72 h post dsRNA injection into hemocoel and observed the variation in the fat body.

To address the reason of metamorphosis failure, we observed the fat body PCD after *Klf15* knockdown. The fat body remained intact and consisted of soft flaky tissue during feeding (from 6th-24 h to 48 h). At the 6th-72 h, the fat body began to decompose and was severely dissociated at the 6th-120 h. Nile Red staining showed that the LD in the fat body cells were densely distributed during feeding period from 6th-24 h to 48 h and broken down to small droplets after entering the metamorphic phase. Terminal deoxynucleotidyl transferase (TdT) dUTP nick-end labeling (TUNEL) staining revealed that fat body apoptosis occurred after larvae entered metamorphosis at the 6th-72 h and enhanced at the 6th-120 h (Fig 3A). The transformation of LC3-I (microtubule-associated protein 1 light chain 3, also known as autophagy-related 8, ATG8) to LC3-II (phosphatidyl ethanolamine form), which indicates autophagy [31], and cleaved-caspase 3 (CASP3), the indicator of apoptosis [32], were analyzed using western blotting to examine the autophagy and apoptosis in the fat body. LC3-II appeared at the 6th-24 h and lasted to 6th-120 h, and disappeared at pupal stage. The cleaved-CASP3 appeared later than LC3-II at the 6th-72 h and continued into pupal stage (Fig 3B and 3Bi), indicating the sequential occurrence of autophagy and apoptosis in the larval fat body during metamorphosis. However, after knockdown of *Klf15*, the fat body cells remained intact, LD were kept large and dense, and the TUNEL signal was not detected (Fig 3C). The transmission electron microscopy (TEM) showed that the number of typical autophagosomes, which had two or single-membraned and contained degenerating cytoplasmic organelles or degraded LD, and autophagic vacuoles [33], decreased in the fat body after *Klf15* knockdown compared with the *dsGFP* control. Furthermore, the apoptotic nuclei that manifested as swelling, chromatin pyknosis and deepening of staining were repressed in the *dsKlf15* group, compared to the cells in the *dsGFP* (Fig 3D). The statistical analysis validated the results (Fig 3Di and 3Dii). Western blotting further revealed that the formation of LC3-II and cleaved-CASP3 were decreased by *Klf15* knockdown (Fig 3E). The data indicated that KLF15 promotes both autophagy and apoptosis in the fat body.

**Fig 4:**
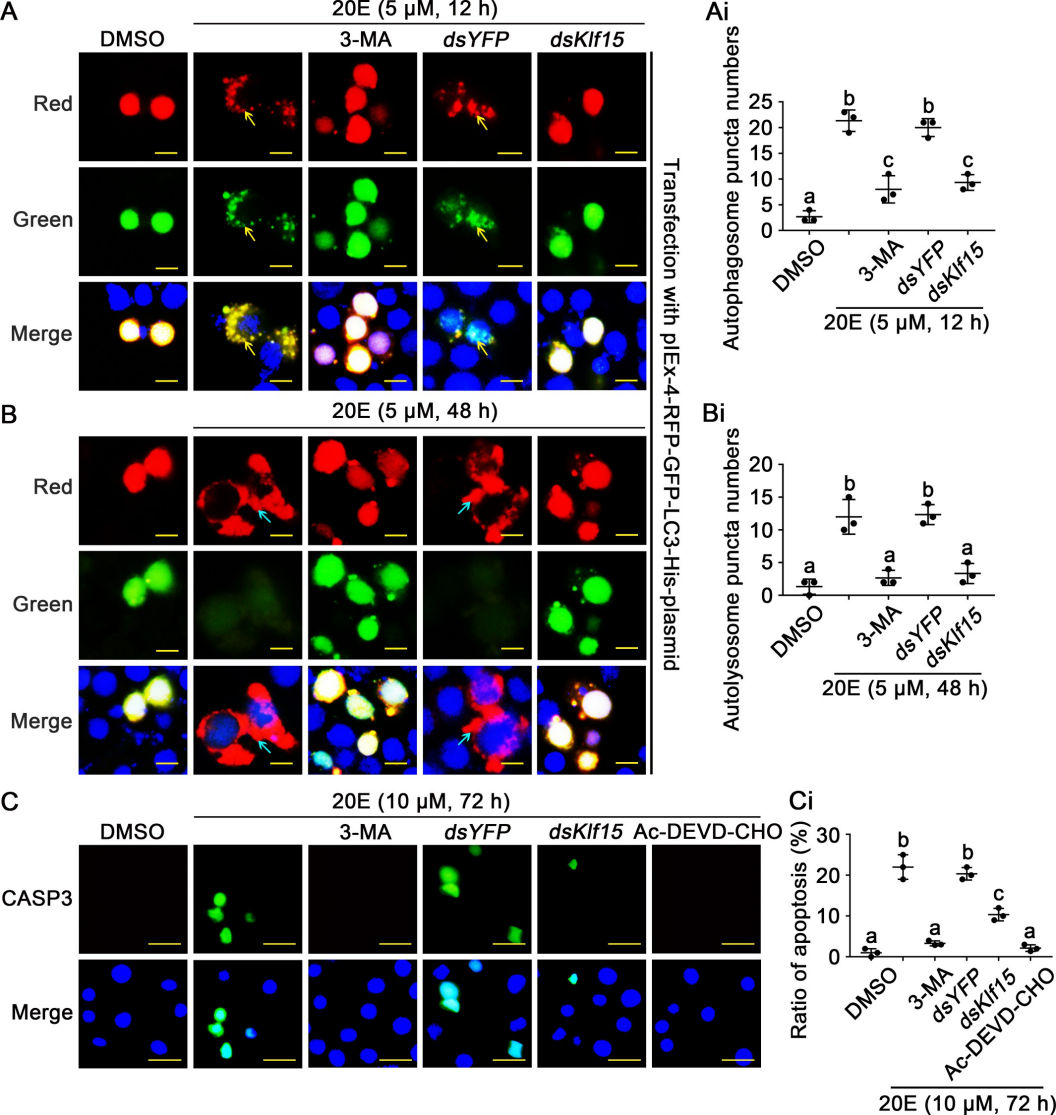
KLF15 promoted autophagy and apoptosis as detected by autophagic flux and CASP3 activity. **A and B.** The autophagic flux were detected in HaEpi cells when *Klf15* was knocked down, *dsYFP* was used as the control. 3-MA (10 μM) is an autophagosome formation inhibitor; the yellow bars represented 20 μm. The yellow arrows represented autophagosome puncta. The blue arrows represented autolysosome puncta. **Ai and Bi.** Counted the number of autophagosome puncta and autolysosome puncta in successfully transfected with pIEx-GFP-RFP-LC3 cells. **C.** After knocked down *Klf15* in HaEpi cells, examination of apoptosis by the addition of active CASP3, Ac-DEVD-CHO is a CASP3 inhibitor, the bars represented 50 μm. **Ci.** Quantification apoptotic cells in total from C.

The role of KLF15 in 20E-mediated autophagic flux were further examined by overexpressing pIEx-4-RFP-GFP-LC3-His fluorescent double-labeled plasmid in HaEpi cells. The cells kept intact in DMSO solvent control. 20E induced puncta of autophagosome appearance in 12 h. The autophagy inhibitor 3-Methyladenine (3-MA) inhibited autophagy. *dsYFP* control did not change 20E-induced autophagy, however, knockdown of *Klf15* blocked the formation of autophagosomes (Fig 4A and 4Ai). 48 h later, similar results were observed, in addition to the green fluorescence disappeared in autophagosomes (Fig 4B and 4Bi), showing the autophagic flux from autophagosome to autolysosome, and knockdown of *Klf15* blocked these processes. Furthermore, knockdown of *Klf15* also resulted in a significant decrease in CASP3 activity indicated by green fluorescence under 10 μM 20E stimulation for 72 h. Autophagy inhibitor 3-MA and apoptosis inhibitor Ac-DEVD-CHO also prevented 20E-induced apoptosis (Fig 4C and 4Ci), suggesting 20E-induced apoptosis relied on autophagy. The interference efficiency was showed (S5 Fig). Flow cytometry revealed that *dsYFP* plus 20E induced 13–19% cell apoptosis, whereas *dsKlf15* plus 20E induced only 8–9% cell apoptosis (S6A and S6Ai Fig). The Cell Counting Kit-8 (CCK-8) assay was used to confirm the role of KLF15 in 20E-induced cell death. Results showed that 10 μM 20E significantly decreased cell viability, however, cell viability increased after *Klf15* knockdown compared with *dsYFP* control (S6B Fig). All these data suggested that KLF15 was involved in 20E-induced autophagy and apoptosis.

**Fig 5:**
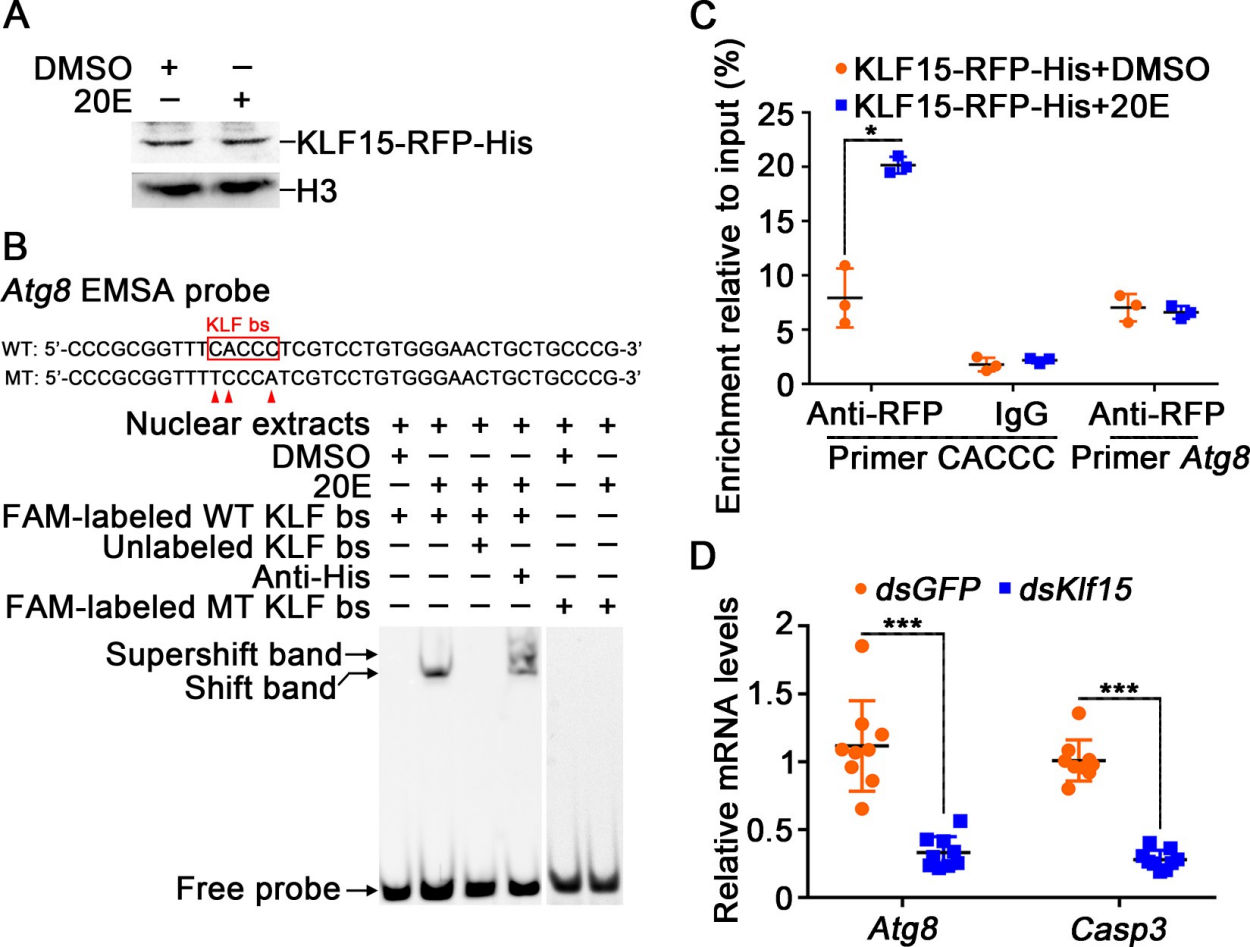
KLF15 promoted autophagy through upregulating *Atg8*. A. Nuclear proteins from KLF15-RFP-His overexpressed cells were extracted for EMSA. **B.** KLF bs on the *Atg8* promoter bound to KLF15 detected by EMSA assay. WT and MT represent KLF bs probe and KLF bs mutant probe, respectively. **C.** ChIP assay showing 20E promoted *Atg8* expression via KLF15 binding to KLF bs. Primer CACCC targeting KLF bs. Primer *Atg8* targeting *Atg8* ORF. **D.** Changes in the expression levels of *Atg8* and *Casp3* after *Klf15* knockdown.

To demonstrate the mechanism that KLF15 promoting autophagy and apoptosis, we analyzed the regulation of KLF15 on the transcription of the PCD-related genes. We did not find KLF bs in the *Casp3* promoter, however, we found KLF bs 5′-CACCC-3′ in the promoter of *Atg8* based on the KLF bs in humans [34, 35]. KLF15-RFP-His was thus overexpressed in cells and an EMSA demonstrated that the overexpressed KLF15-RFP-His in the nuclear extracts of HaEpi cells could bind to the CACCC containing probe designed from *Atg8* promoter, after point mutation of KLF bs of *Atg8* [10, 36], the probe was no longer bound by nuclear proteins (Fig 5A and 5B). ChIP experiment proved that KLF15-RFP-His bound the CACCC fragment of *Atg8* under 20E induction (Fig 5C). Knockdown of *Klf15* in larvae decreased *Atg8* and *Casp3* expression (Fig 5D). In addition to *Atg8*, knockdown of *Klf15* also suppressed the expression of *Atg4b*, *Atg12*, and *Atg14* (S7 Fig). All data suggested that 20E regulates autophagy and apoptosis via KLF15, which upregulating *Atgs* transcription.

### Autophagy and apoptosis produced substrates for gluconeogenesis

**Fig 6:**
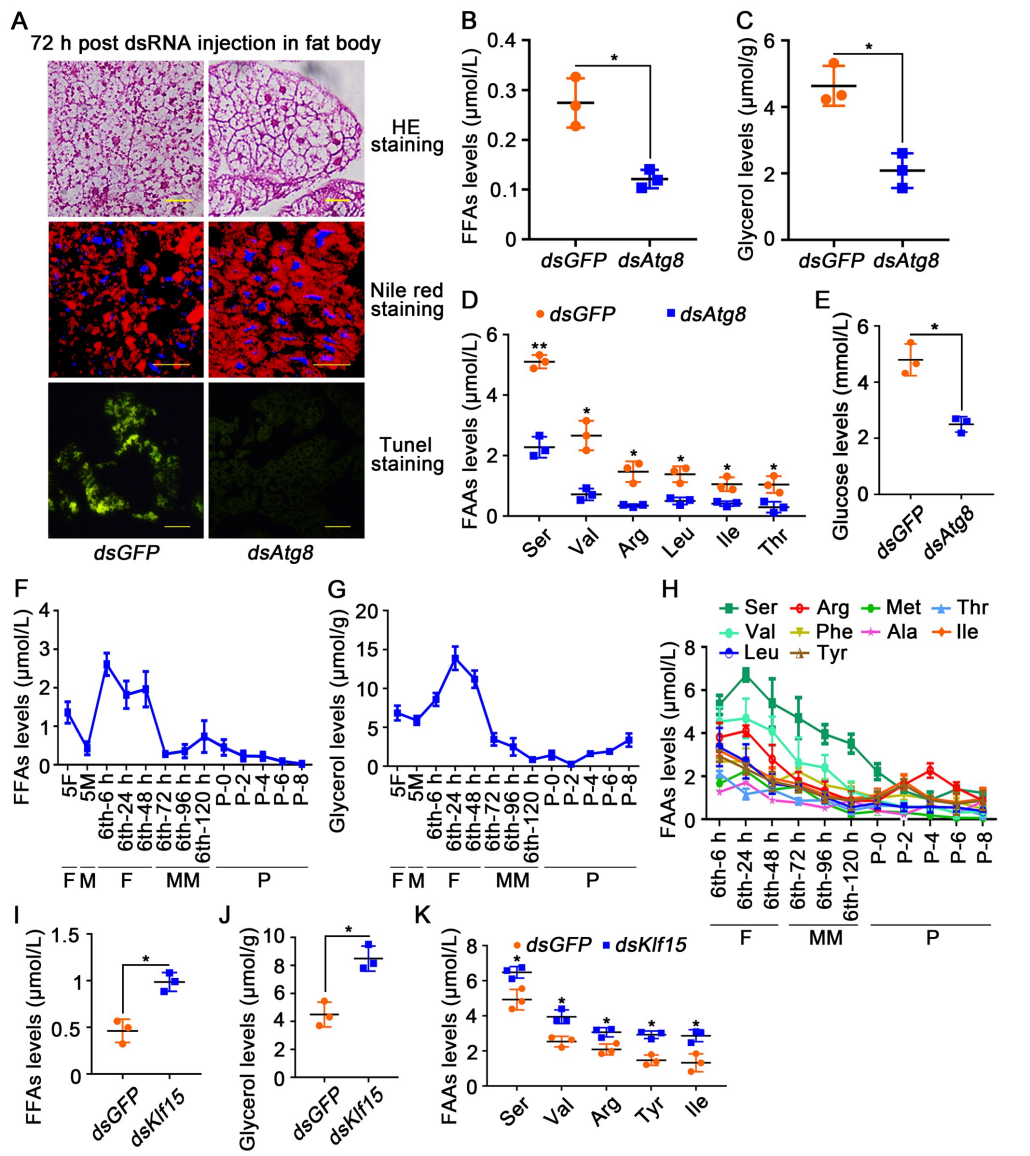
Autophagy presented substrates for gluconeogenesis. **A.** HE staining, Nile red staining and TUNEL staining showing the morphology of fat body after *dsGFP* and *dsAtg8* injection into hemocoel. **B and C.** Levels of FFAs in the hemolymph and glycerol in the fat body after knockdown of *Atg8*. **D.** FAAs levels in the hemolymph after knockdown of *Atg8*. **E.** Glucose levels in the hemolymph decreased after knockdown *Atg8*. **F and G.** FFAs levels in the hemolymph and glycerol levels in the fat body from 5th instar larvae to 8-day-old pupae. **H.** FAAs levels in the hemolymph. **I and J.** FFAs and glycerol levels after first injection *dsKlf15* for 72 h. **K.** FAAs levels after knockdown of *Klf15*.

To determine the relationship between autophagy and gluconeogenesis, *dsAtg8* was injected into the 6th-6 h larvae to block autophagy. Compared with *dsGFP*, the fat body was not dissociated, the LD was not broken, and the apoptosis was not observed after knockdown of *dsAtg8* (Fig 6A). Moreover, the levels of FFAs and glycerol, the degraded products of fat body, were lower compared to *dsGFP* (Fig 6B and 6C). The levels of certain FAAs, including serine (Ser), valine (Val), arginine (Arg), leucine (Leu), isoleucine (Ile) and threonine (Thr), which have the potential to be metabolized to produce glucose via gluconeogenesis [36,37], exhibited significantly reduction after *dsAtg8* knockdown (Fig 6D). The hemolymph glucose levels also decreased after knockdown of *dsAtg8* (Fig 6E). Knockdown of *Casp3* also inhibited fat body degradation and decreased glucose levels (S8 Fig). These results suggested that autophagy and apoptosis generate substrates for gluconeogenesis to produce glucose.

In addition, FFAs, glycerol and all FAAs levels were high during the feeding period from 6th-6 h to 48 h, but gradually decreased during metamorphosis (Fig 6F–6H), suggesting that FFAs, glycerol and FAAs were used for gluconeogenesis during metamorphosis. Knockdown of *Klf15*, FFAs (Fig 6I), glycerol (Fig 6J) and FAAs (Ser, Val, Arg, Tyr, Ile) (Fig 6K) were accumulated, indicating the role of KLF15 in the metabolism of these substrates in gluconeogenesis.

### Glycometabolism was changed from glycolysis to gluconeogenesis during insect metamorphosis

**Fig 7:**
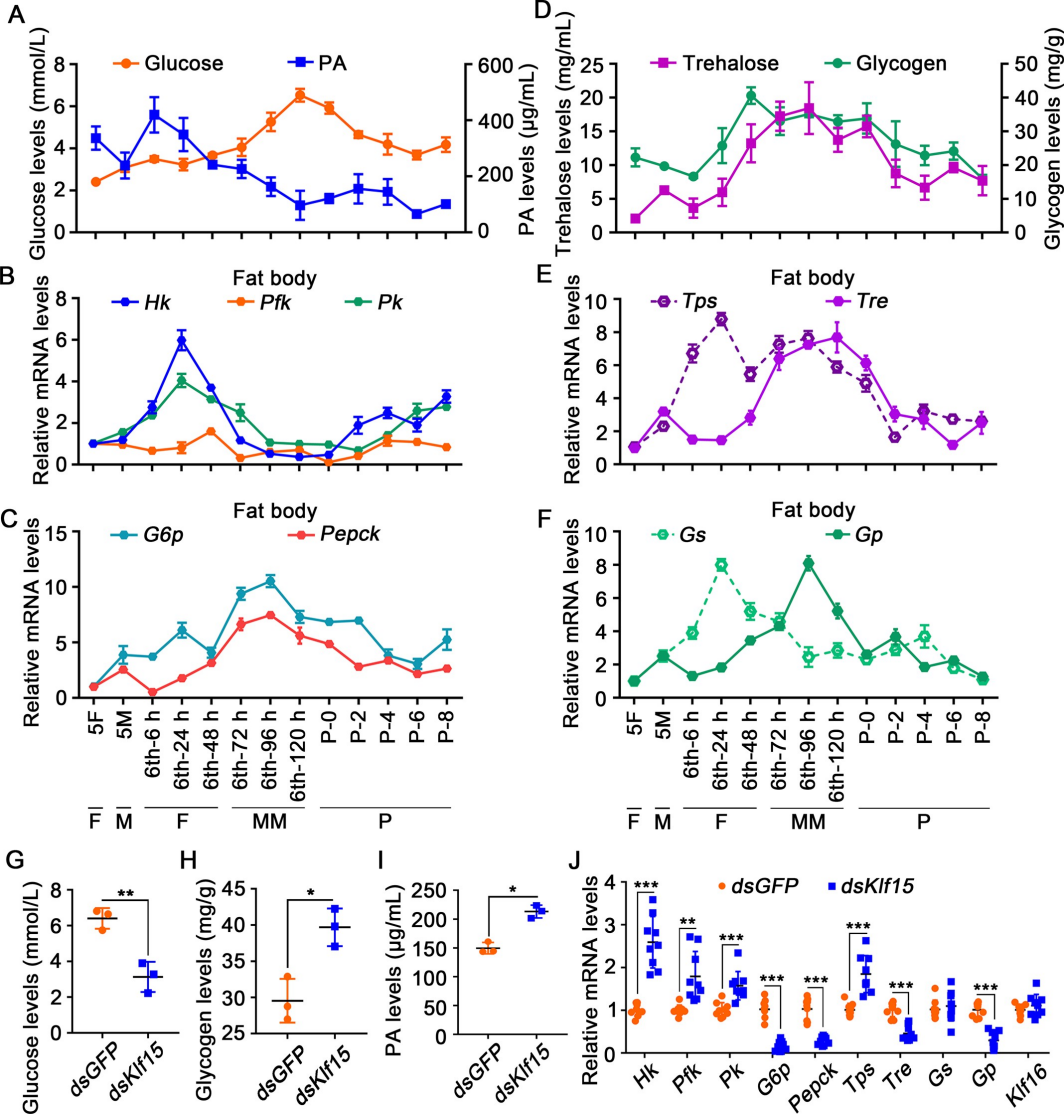
Variation in the levels of metabolites and related genes expression after *Klf15* was knocked down in larvae. **A.** The levels of glucose and PA in the hemolymph. **B.** The expression profiles of *Hk*, *Pfk*, and *Pk* in the fat body. **C.** The expression profiles of *G6p* and *Pepck* in the fat body. **D.** Trehalose levels in the hemolymph and glycogen levels in the fat body. **E.** qRT-PCR showing the mRNA expression profiles of *Tps* and *Tre* in the fat body. **F.** The expression profiles of *Gs* and *Gp* in the fat body. **G-I.** Variation in the levels of metabolites after first injection *dsKlf15* for 72 h. **J.** Changes in the expression levels of certain genes after *Klf15* knockdown by injection of dsRNA, as detected using qRT-PCR.

To assess changes in glycometabolism during development, we measured glucose and PA levels in the hemolymph. Glucose levels in larval hemolymph increased from 6th-72 h wandering stage and peaked at the 6th-120 h metamorphosis stage. PA levels were highest during the 6th-6 h to 24 h feeding stage and rapidly decreased after the 6th-48 h stage (Fig 7A), suggesting glucose not only could not be metabolized to PA by glycolysis, but also increased probably by gluconeogenesis after feeding cease.

Therefore, the expression levels of genes encoding key enzymes in glycolysis and gluconeogenesis were further examined to confirm the variation of glycolysis and gluconeogenesis. The mRNA encoding hexokinase (*Hk*), and pyruvate kinase *(Pk*), which are the key enzymes that catalyze irreversible reactions in the glycolysis pathway, were highly expressed at the 6th-24 h during the feeding period; however, their expression levels decreased during metamorphosis, with some increased after the mid-pupa stage (Fig 7B). In contrast, the mRNA levels of the key enzymes for gluconeogenesis, *G6p* and *Pepck*, were highly increased during metamorphosis (Fig 7C). These data confirmed the transformation of glycolysis to gluconeogenesis during metamorphosis, indicating that active glycolysis during the feeding period promotes glucose metabolism and an increase in PA levels, whereas during the metamorphosis phase, gluconeogenesis accelerates the utilization of PA and leads to an increase in glucose.

We also measured trehalose levels in the hemolymph and glycogen levels in the fat body to determine the source of high glucose levels during metamorphosis. We found trehalose increased from the 6th-48 h feeding stage and reached a peak at the 6th-96 h wandering stage, then decreased rapidly. Glycogen levels continued to rise during the feeding period from the 6th-24 h to 48 h, and then decreased after feeding stopped from the 6th-72 h larvae to pupae (Fig 7D), indicating that high levels of glucose may result from the degradation and no longer synthesis of trehalose and glycogen. To support the above hypothesis, the mRNA expression levels of trehalose-phosphate synthase (*Tps*), which synthesizes trehalose, and trehalase (*Tre*), which degrades trehalose, were examined in the fat body. *Tps* showed high expression during the feeding period at the 6th-24 h, and then declined during metamorphosis. By contrast, *Tre* showed low expression at the 6th-24 h feeding stage, but was highly expressed during metamorphosis (Fig 7E), supporting that glucose levels are increased during metamorphosis by decreasing trehalose synthesis and increasing its degradation simultaneously. Glycogen synthase (*Gs*) expression in the fat body increased from the 6th-6 h to 24 h feeding stage, and decreased during metamorphosis after feeding stopped. The expression levels of the gene encoding *Gp*, which degrades glycogen, increased at the 6th-96 h and decreased during the pupal stage (Fig 7F), indicating that a decrease in glycogen synthesis and an increase in glycogen degradation also contributed to the elevated glucose levels during metamorphosis. After *Klf15* was knocked down, glucose levels dropped significantly (Fig 7G), trehalose without significant increased (S9 Fig), whereas the levels of glycogen accumulated (Fig 7H), and PA increased (Fig 7I).

The expression levels of genes involved in glycometabolism were examined to determine the mechanism by which KLF15 regulated glycometabolism reprogramming. After knocking down *Klf15*, the mRNA levels of *G6p* and *Pepck* for gluconeogenesis, *Tre* for trehalose degradation, and *Gp* for glycogen degradation were decreased. In contrast, the mRNA levels of *Hk*, *Pfk* and *Pk* for glycolysis, and *Tps* for trehalose synthesis were increased. *Klf16* expression was detected to show that there was no off-target effect of *Klf15* knockdown (Fig 7J). These data suggested that KLF15 promotes glycogen degradation and gluconeogenesis by upregulating the related genes expression.

### KLF promoted *Pepck* expression to produce glucose for metamorphosis

**Fig 8:**
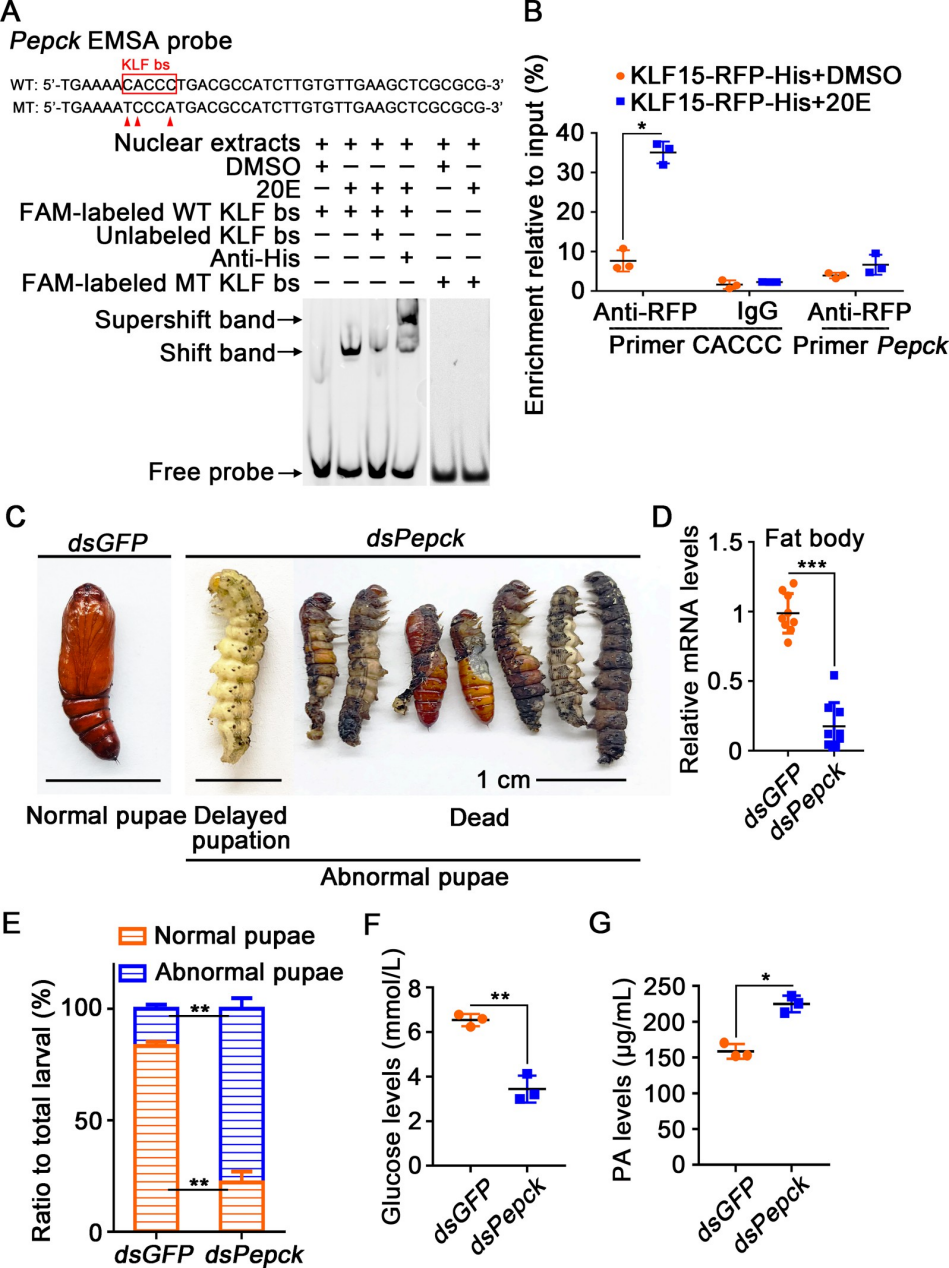
Knockdown of *Pepck* delayed pupation and decreased hemolymph glucose levels. **A.** KLF bs on the *Pepck* promoter bound to KLF15 detected by EMSA assay. **B.** ChIP assay showing 20E promoted *Pepck* expression via KLF15 binding to KLF bs. **C.** Phenotypes after injection of *dsPepck* and *dsGFP* from 6th-6 h to 72 h; the ruler represents 1 cm. **D.** qRT-PCR validation of the interference efficiency in the fat body after the third dsRNA injection. **E.** Ratio of phenotypes. **F.** Glucose levels in the hemolymph decreased after knockdown *Pepck*. **G.** PA levels in the hemolymph increased after knockdown *Pepck*.

PEPCK is the first rate-limiting enzyme in the gluconeogenesis pathway, catalyzing the synthesis of phosphoenolpyruvate from oxaloacetate. The activity of PEPCK is primarily regulated at the transcription level, and the gene’s expression directly affects gluconeogenesis rate [37]. We found KLF bs in the promoter region of *Pepck* and designed an EMSA probe containing KLF bs to determine regulation of KLF15 to *Pepck* transcription. The formation of a normal shift band was observed after incubating the labeled probe with 20E-induced nuclear proteins that containing overexpressed KLF15-RFP-His, and could be competitively decreased by the unlabeled probe. Meanwhile, a supershift band formed when the antibody against His-tag, which identified the overexpressed KLF15-RFP-His, was added to the reaction mixture. The point mutation of KLF bs inhibited the binding of the probe to nuclear proteins (Fig 8A). ChIP experiment proved that 20E promoted the binding of KLF15 to CACCC motif of *Pepck* to increase the expression of *Pepck* (Fig 8B). These data revealed that KLF15 promotes gluconeogenesis by upregulating the *Pepck* transcription.

To directly demonstrate that gluconeogenesis promotes glucose accumulation, we knocked down *Pepck* and found that the larvae failed metamorphosis (Fig 8C–8E). The glucose levels in the hemolymph were decreased significantly (Fig 8F), and PA appeared to accumulate (Fig 8G), confirming the role of PEPCK in gluconeogenesis and insect metamorphosis.

pIEx-4-RFP-His and pIEx-4-KLF15-RFP-His were overexpressed in HaEpi cells separately (S10A and S10B Fig), and superimposed 20E stimulation. Compared with RFP-His, overexpression of KLF15-RFP-His significantly promoted *Atg8*, *Casp3* and gluconeogenesis-related genes, *G6p* and *Pepck*, transcription (S10C Fig).

## Discussion

The regulatory mechanism of glycometabolism homeostasis is fascinating and mysterious. The present study revealed that the insect molting hormone 20E via KLF15 regulates glycometabolism reprogramming from glycolysis to gluconeogenesis and promotes fat body autophagy and apoptosis to supply substrates for gluconeogenesis during metamorphosis.

### 20E reprogrammed glycometabolism from glycolysis to gluconeogenesis

Various hormones regulate glycometabolism. In humans, insulin maintains blood glucose levels via promoting the uptake of glucose from blood, utilizing glucose by glycolysis, increasing the synthesis and storage of glycogen in tissue cells [38], accelerating the aerobic oxidation of glucose, and inhibiting gluconeogenesis by limiting the release of substrates from storage, such as adipose tissue. Insulin like peptides (ILPs) play similar roles in insects via insulin/insulin-like growth factor (IGF) signaling (IIS) [39]. In feeding conditions, insulin promotes glucose uptake into cells via glucose transporter 4 (GLUT4) [40]. In the clinical treatment of human diseases, steroid hormones, such as glucocorticoids, repress insulin function and increase blood glucose levels. The insect hormone 20E represses larval feeding by competing for binding with the dopamine receptor (DopEcR) [41], and antagonizes IIS to elevate hemolymph glucose levels by repressing the expression and phosphorylation of the receptor during metamorphosis [26]. The glucose levels increased to a peak before pupation and then decreased after the mid-pupa period, which appeared similar to the curve of 20E titer during metamorphosis [22]. In the present study, we showed that 20E represses glycolysis and the synthesis of glycogen and trehalose by repressing the related genes expression to block the metabolic export of glucose in the hemolymph. On the other hand, 20E promotes the degradation of glycogen, trehalose and gluconeogenesis, which increases the metabolic import of glucose in the hemolymph. The levels of PA also continued to decrease after the larvae stop feeding. The decrease in PA is due to 20E counteracting insulin to suppress glycolysis during metamorphosis, as well as a 20E-mediated increase in gluconeogenesis. Taken together, 20E regulates the reprogramming of glycometabolism from glycolysis to gluconeogenesis to increase glucose during metamorphosis.

Transcriptional regulation links changes in a cell’s internal status to how it perceives and reacts to external stimuli [42, 43]. Variations in transcriptional regulation, such as transcription factors, chromatin modifications, or transcription factor co-regulators, alter cellular signaling pathways and initiate metabolic reprogramming to satisfy the needs of fast cell proliferation, differentiation and death [44–46]. In metabolic pathways, there are a variety of enzymes encoded by distinct genes, and their promoters may have different transcription factor binding sites, resulting in diverse transcriptional regulation and thus altering the metabolic process. In mammals, KLF15 is closely related to metabolism and highly expressed in the fasting period, thus playing an important role in gluconeogenesis [11]. KLF15 promotes the expression of a number of enzymes involved in AAs catabolism, including alanine aminotransferase 1 (ALT1), proline dehydrogenase (PRODH), and tryptophan 2, 3-dioxygenase (TDO2) to provide carbon substrates for the formation of nutrients [12, 13]. The gene encoding fasting-induced acyl-CoA synthetase short chain family member 1 (ACSS1) and other fasting-induced genes in skeletal muscle are mainly regulated by KLF15 [6]. The *Klf15* gene is a direct transcriptional target of the glucocorticoid receptor (GR), participating in glucocorticoid-regulated physiological processes in mammals [7]. However, how KLF15 regulates glucose metabolism reprogramming and related mechanisms have not been reported. Our research showed that *Klf15* is highly expressed by 20E regulation. 20E, via KLF15, increases the expression of a set of genes to promote the degradation of glycogen and trehalose, fat body autophagy and apoptosis and gluconeogenesis.

### 20E, via KLF15, promotes genes expression for autophagy

Autophagy is a common mechanism for energy supply in eukaryotic cells during nutrient shortage. It degrades and recycles self-protein, organelles or invading pathogens in cells through the lysosome pathway [47]. Autophagy is closely related to nutritional status. When cell nutrition is lacking, cells immediately initiate autophagy to maintain the balance of the AA pool in the cytoplasm. Human KLF15 inhibits the expression of cell cycle regulatory protein, cyclin D and E, and cyclin-dependent kinase 2 (CDK2), to inhibit glomerular mesangial cells entering the S phase in the cell cycle, thereby inhibiting cell proliferation [48]. KLF3 in *C*. *elegans* and KLF4 in *H*. *sapiens* promote autophagy, and KLF3 promotes *Atg2*, *Atg9* and *Atg16* expression [9]. We found a KLF bs in the *Atg8* promoter, proved that KLF15 directly promoted *Atg8* transcription. We also found other *Atgs* (*Atg3*, *Atg4b*, *Atg7*, *Atg12* and *Atg14*) had the KLF bs in their promoters and knockdown of *Klf15* inhibited the expression of *Atg4b*, *Atg12*, and *Atg14*. KLF15 also increased the expression of the genes involve in glycogen and trehalose degradation, gluconeogenesis and apoptosis. Therefore, KLF15 in *H*. *armigera* is a transcription factor that promotes the occurrence of autophagy and apoptosis. KLFs have distinct domains at amino-terminal regions, which determine their transcriptional properties [49]. While KLF bs are same, KLFs may be expressed in different tissues; on the other hand, they may compete for the same binding site [50, 51]. Besides KLF15, the *H*. *armigera* genome contains other KLFs, which need to be study on their functions.

### Fat body autophagy and apoptosis contribute substrates for gluconeogenesis

Autophagy is closely related to apoptosis [52, 53]. A lack of nutrients causes autophagy, which is followed by apoptosis in mouse neuronal cells. The application of the autophagy inhibitor 3-MA inhibits the release of cytochrome C and the activation of caspase [54]. During the development of holometabolous insects, most autophagy-related genes are expressed before apoptosis-related genes. High titer 20E increases intracellular calcium ion levels, allowing ATG5 to be cleaved, activating CASP3 and promoting apoptosis [25]. 20E induces CTSD (cathepsin D) maturation through autophagy, which promoting CASP3 cleavage and apoptosis in the midgut [22].

The primary nutrients in LD are triglycerides, and the products of triglyceride decomposition are FFAs and glycerol [55, 56]. Glycerol is converted directly into metabolic intermediates to produce glucose, while FFAs are converted into acetyl-CoA via the β-oxidation pathway to enter the TCA cycle for energy supply. Under starvation conditions, the conversion of glycogenic AAs produced by hepatic autophagy into glucose via gluconeogenesis has been considered a potential metabolic contributor in mice [57, 58]. We found autophagy promotes the continuous breakdown of LD as metamorphosis develops after larvae stop feeding, and knockdown of *Atg8* resulting in a decrease of FFAs, glycerol, FAAs and glucose, suggesting autophagy produces various substrates for gluconeogenesis. However, the gluconeogenesis promotes the consumption of FFAs, glycerol, FAAs to produce glucose directly or indirectly, therefore FAAs, FFAs and glycerol cannot be accumulated during metamorphosis.

KLF15 can directly and positively regulate *Atg8* and *Pepck* expression, therefore, autophagy and gluconeogenesis were both inhibited after *Klf15* knockdown. However, the variation of metabolites FFAs, glycerol and FAAs were opposite after knockdown of *Atg8* and *Klf15*, that knockdown of *Atg8* decreased FFAs, glycerol and FAAs, but knockdown of *Klf15* increased these metabolites. This is due to knockdown of *Atg8* only blocked autophagy and therefore blocked the coming of the substrates, and the gluconeogenesis was still performed by PEPCK under KLF15 regulation. Whereas, knockdown of *Klf15* blocked not only autophagy and the coming of the substrates, but also blocked gluconeogenesis and the consumption of the substrates by repressing *Pepck*, thus FFAs, glycerol and FAAs were accumulated after balance of the metabolism network *in vivo*.

KLF15 expressed in the fat body and epidermis during metamorphosis. RNAi in insect may produce a systematic effect in various tissues, and the metabolites may be communicated in tissues. However, the fat body is the center of insect metabolism, which stores energy substrates during feeding and produces energy substrates by autophagy and apoptosis during non-feeding or nutrients shortage. Epidermis has not been reported occur autophagy and apoptosis as those in the midgut and fat body, in addition to local cells, which may occur autophagy and apoptosis [22, 59]. Therefore, KLF15 plays key roles in the fat body to integrate autophagy and gluconeogenesis to maintain glucose homeostasis under 20E regulation.

Previous study addressed that high glucose levels during metamorphosis of *H*. *armigera* is due to 20E represses insulin receptor expression and phosphorylation [39], it is similar to the type 2 diabetes (T2D) in humans [60], which means that glucose cannot be absorbed into the cells, therefore increasing hemolymph glucose levels. Here, we found glycolysis was changed to gluconeogenesis during metamorphosis by 20E regulation, which also increases hemolymph glucose. However, the glucose levels decreased after insect entering the pupal stage, suggesting the high levels glucose stored during metamorphosis may become the main energy to be used to support the pupal development. Therefore, the glucose levels are well regulated in physiological regions during insect development.

## Conclusions

**Fig 9:**
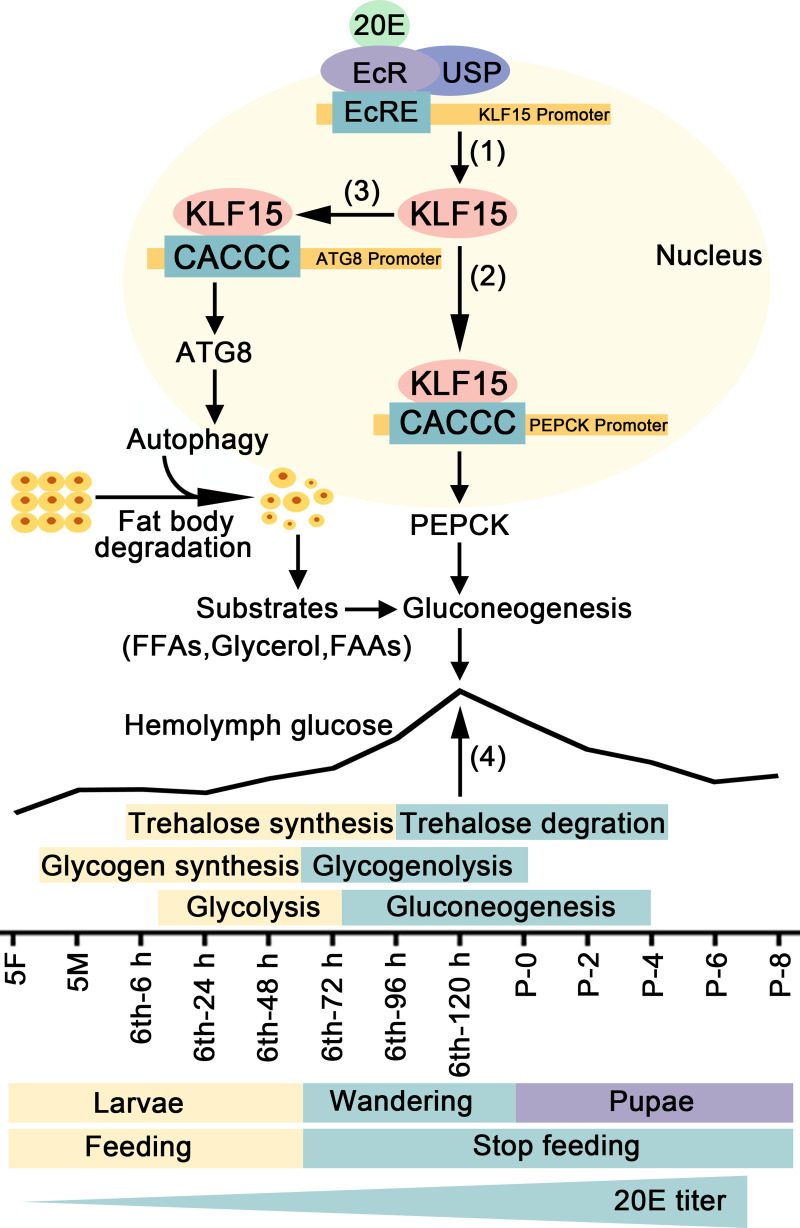
A diagram illustrating 20E regulates glycometabolism reprogramming, autophagy, and apoptosis via KLF15. 20E promotes the expression of *Klf15* via nuclear receptors (1). By binding to the KLF bs, KLF15 promotes the transcription of *Pepck*, a crucial enzyme in gluconeogenesis (2). KLF15 increases the transcription of the autophagy-related gene *Atg8* to induce autophagy (3), thereby providing substrates for gluconeogenesis. The increase of glucose during metamorphosis is due to trehalose and glycogen degradation, glycolysis inhibition, and gluconeogenesis (4).

20E, via KLF15, promoted autophagy, apoptosis, and gluconeogenesis. Autophagy and apoptosis supplied substrates for gluconeogenesis. Glycolysis was changed to gluconeogenesis from feeding to non-feeding during metamorphosis. The elevation of hemolymph glucose levels was the result of blocking of glycolysis, enhancing of degradation of glycogen and trehalose, and increased gluconeogenesis (Fig 9).

## Materials and methods

### Ethics statement

The antibody preparation in rabbit was in accordance with protocols approved by the Animal Care & Welfare Committee at Shandong University School of Life Sciences (SYDWLL-2021-54).

### Experimental animals

*H*. *armigera* were reared on an artificial diet in our laboratory at 27 ± 1°C with 60–70% humidity under a light dark schedule of 14 h:10 h. The larvae were fed with artificial feed composed of soy flour, wheat germ, multivitamins, sucrose, and inorganic salts [61].

### Preparation of rabbit polyclonal antibody against KLF15

A fragment of *Klf15* (nucleotide sequence 1 bp to 564 bp, amino acids 1 to 188) was inserted into plasmid pET-30a. The recombinant plasmid was sequenced and expressed in *Escherichia coli* strain Rosetta (DE3). Recombinant KLF15 protein was formed in inclusion body and was purified after denaturation and refolding. Rabbits were immunized with the purified antigen to obtain rabbit polyclonal antibodies (anti-KLF15 antibodies); the molecular weight of KLF15 is 30 kDa (S11 Fig).

### Western blotting

We selected larvae or pupae at different ages, cut open the body along the midline and rinsed the epidermis, midgut, and fat body separately in PBS, placed the tissues in 400 μL Tris-HCl buffer (pH 7.5, 40 mM) with 4 μL phenylmethylsulfonyl fluoride (PMSF) on ice and ground them fully. The samples were centrifuged at 10000 × *g* for 15 min, and the supernatant was retained and incubated in a 100°C water bath for 15 min. Protein (20 μg) was separated by sodium dodecyl sulfate-polyacrylamide gel electrophoresis (SDS-PAGE) and then transferred onto a nitrocellulose membrane. The membrane was incubated in blocking solution prepared with TBST (0.02% tween in TBS) for 1 h at room temperature. The primary antibodies were added into the blocking buffer and incubated overnight at 4°C. The polyclonal antibodies against *H*. *armigera* LC3 and KLF15 were produced in our laboratory and were diluted in 5% skim milk at 1:100. The antibodies against *H*. *armigera* β-actin and cleaved-CASP3 were diluted in 5% skim milk at 1:500–1:5000. After being washed two times with TBST for 10 min each time, the membrane was incubated with the diluted secondary antibodies (ZB-2301/ZB-2308, ZSGB-BIO, Beijing, China) labeled with horseradish peroxidase/alkaline phosphatase (1:7000 in the blocking buffer) for 2 h at room temperature. The membrane was washed twice with TBST for ten min each time, followed by TBS (pH 7.5, 150 mM NaCl, 10 mM Tris-HCl) for five min once. The membrane was immersed in High-sig ECL Western Blotting Substrate (180–501, Tanon Science & Technology, Shanghai, China) and the immunoreactive protein bands were visualized using the ECL luminescence method. The protein bands were detected using a 5200 Chemiluminescence Imaging System (Tanon Science & Technology). The immunoreactive protein bands were analyzed using Image J software (NIH, Bethesda, MN, USA).

### Hormone stimulation of *Klf15*

20E (16145, Cayman Chemical, Ann Arbor, USA) was diluted to 10 mg/mL with DMSO for storage. When conducting experiments, 20E was diluted 100-fold with sterile phosphate buffered saline (PBS, pH 7.4, 10 mM Na_2_HPO_4_, 1.8 mM KH_2_PO_4_, 140 mM NaCl, and 2.7 mM KCl). We selected three 6th-6 h larvae and injected them with 20E at 100, 200, and 500 ng, respectively; cultured them for 1, 3, 6, 12, and 24 h; and total RNA was extracted for qRT-PCR. The control groups were treated with the same amount of DMSO and processing time.

### qRT-PCR

Total RNA was extracted from the larvae and pupae using the Trizol reagent (O10820, TransGen Biotech, Beijing, China) and then detected the RNA concentration, quantified 2 μg RNA transcribed to single stranded cDNA using a cDNA synthesis kit (G492, Abm, Richmond, Canada). qRT-PCR was performed in a final volume of 10 μL, containing 5 μL of TransStart Tip Green qPCR Supermix (291829AX, Aidlab, Beijing, China), 1 μL of cDNA (1:7 dilution), and 2 μL each of the forward and reverse primers (1 μM) (sequences showed in S1 Table), the β-actin gene (*Actb*) was used as the internal reference. Data were analyzed by the formula R = 2^– (ΔCt sample–ΔCt control)^, representing the relative transcription levels, ΔCt is the difference between the cycle threshold (Ct) of the genes and the average *Actb* transcript levels in the experimental or control sample.

### EMSA

Nuclear proteins were extracted from HaEpi cells expressing EcR and KLF15 using a nuclear extract kit (R0050, Solarbio, Shanghai, China) according to the manufacturer’ s protocol. After 6 h induction with DMSO or 20E (5 μM), nuclear proteins were extracted. Fluorophore 6-carboxy-fluorescein (FAM)-labeled probes (sense 5’- TCTCAATTACGTTCAATAAACGGCTTTGTTAA-3’ and antisense 5’- TTAACAAAGCCGTTTATTGAACGTAATTGAGA-3’) used in EMSA from the EcRE fragment of *Klf15*, (sense 5’-TCTCAATTACATGTAATAAACGGCTTTGTTAA-3’ and antisense 5’-TTAACAAAGCCGTTTATTACATGTAATTGAGA-3’) were mutation probe; (sense 5’-CCCGCGGTTTCACCCTCGTCCTGTGGGAACTGCTGCCCG-3’ and antisense 5’-CGGGCAGCAGTTCCCACAGGACGAGGGTGAAACCGCGGG-3’) used in EMSA from the KLF bs of *Atg8* and (sense 5’-TGAAAACACCCTGACGCCATCTTGTGTTGAAGCTCGCGCG-3’ and antisense 5’- CGCGCGAGCTTCAACACAAGATGGCGTCAGGGTGTTTTCA-3’) used in EMSA from the KLF bs of *Pepck*, (sense 5’-CCCGCGGTTTTCCCATCGTCCTGTGGGAACTGCTGCCCG-3’ and antisense 5’- CGGGCAGCAGTTCCCACAGGACGATGGGAAAACCGCGGG-3’), (sense 5’- TGAAAATCCCATGACGCCATCTTGTGTTGAAGCTCGCGCG-3’ and antisense 5’- CGCGCGAGCTTCAACACAAGATGGCGTCATGGGATTTTCA-3’) were mutation probe (produced by the Sangon Company). The probes were dissolved in the annealing buffer (pH 7.5, 10 mM Tris, 50 mM NaCI, 1 mM EDTA). The mixture was heated at 95°C for 10 min and slowly cooled to room temperature. About 15 μg of extracted protein in binding buffer (pH 7.5, 10 mM TrisHCl, 1 mM MgCl_2_, 40 mM KCl, 0.1 mg/ml BSA, 5% glycerol) was incubated with 200 fmol of FAM-labeled probes at room temperature for 15 min [62, 63]. An antibody for His (1.5 μg) was added to the binding reaction in the supershift experiment. A 50-fold amount of unlabeled probe was added for competition experiments. The protein-DNA samples were then run on a 6% polyacrylamide gel at 80 V for 70 min in 0.5 × TBE buffer (NA0036, LEAGENE, Beijing, China). Tanon Gel Imager System was used for imaging and data analysis.

### ChIP

We used 5 μg EcR-RFP-His transfected into HaEpi cells for 72 h (the number of cells is about 8×10^5^−10×10^5^), which were then treated with 5 μM 20E for 6 h to detect the binding of EcR in the promoter of *Klf15*. For KLF bs of *Atg8* and *Pepck* detection, 5 μg KLF15-RFP-His were transfected into HaEpi cells for 72 h, and then treated with 5 μM 20E for 6 h. The cells were treated according to the instructions of the ChIP assay kit (P2078, Beyotime Biotechnology, Shanghai, China). Anti-His antibody was used to detect EcR-RFP-His and KLF15-RFP-His, and IgG was used as a negative control.

### RNA interference (RNAi) in larvae

Long double-stranded RNA (dsRNA) can be used in insects and is broken down into several smaller dsRNA *in vivo* to degrade the transcript of the targeted gene [64]. Using the target sequence as a template, an RNAi kit (AM1626, Thermo Fisher Scientific, Waltham, MA, USA) was used to synthesize dsRNA according to the instructions of the manufacturer. For RNAi knockdown of genes in larvae, 30 larvae at the 6th-6 h were injected with 3 μg of dsRNA into the hemocoel, three times at 24 h apart. The control larvae were treated with the same amount of *dsGFP*. The interference efficiency, key enzymes of the gluconeogenesis pathway, hemolymph glucose levels, and the morphology of the fat body were determined after first injection dsRNA for 72 h. Each RNAi was performed independently three times, using a total ninety insects.

### Fat body morphology observation

Took fresh fat body and embed it in paraffin (Servicebio, Wuhan, China). The paraffin-embedded fat body were cut into 4 μm pieces and fixed on a glass slide before being dried at 60°C for 2 h and stained according to the YF488 TUNEL assay apoptosis detection instructions (T6013, US EVERBRIGHT INC, Suzhou, China). Servicebio company performed the HE staining, Nile red staining and TEM images (S12 Fig). We used an Olympus BX51 fluorescence microscope (Olympus Optical Co., Tokyo, Japan) to observe the fat body morphology and chose the appropriate magnification to take photos and statistics.

### Cell culture

The HaEpi cell line was established from the epidermis of 5th instar larvae in our laboratory [65]. Cells were developed in cell culture flasks loosely attached monolayer and maintained at 27°C. 5 mL Grace’s medium (11300–043, Gibco, USA) and 10% heat-inactivated fetal bovine serum (FBS) (16140063, Gibco, USA) were added to each culture flask and subcultured once a week. We transferred the cells with medium to a 6 or 24-well plate, added 20E for different time stimulation, and used equal diluted DMSO as a control when monitoring autophagic flux, CASP3 activity and flow cytometry. A six-well plate contains approximately 8×10^5^−10×10^5^ cells, while a 24-well plate contains approximately 2×10^5^−3×10^5^ cells. We observed the cells using an Olympus BX51 fluorescence microscope and chose the appropriate magnification to take photos and statistics.

### Autophagy detection

LC3-I and LC3-II were detected in a western blot assay with polyclonal rabbit antibody against *H*. *armigera* LC3. RFP-GFP-LC3-His was overexpressed in HaEpi cells using pIEx-4-RFP-GFP-LC3-His fluorescent double-labeled plasmid to determine the autophagic flux by 20E regulation according to our previous study [25]. As GFP fluorescence is sensitive to an acidic pH, it can be compared with red fluorescence to monitor the occurrence of autophagy [66]. We set different time gradients to observe the occurrence of 20E regulated autophagy. After 5 μM 20E induction for 1 h, both green fluorescence and red fluorescence were seen in cells, which were similar to the control group treated with DMSO. However, after 12 h of stimulation with 20E, some small puncta were visible in the cells, which indicated that autophagosomes were forming. When the stimulation time reached 24 h, the puncta still existed, but the green light began to dim. After 48 h of stimulation, only red fluorescence was visible, green fluorescence was quenched (S13A Fig), indicating the formation of acidic autolysosomes and 20E promoted the transition from autophagy to apoptosis. The autophagic flux was determined by counting the number of autophagosome and autolysosome puncta in successfully transfected with pIEx-GFP-RFP-LC3 cells from three different pictures.

### Apoptosis detection

The rabbit polyclonal anti-cleaved-CASP3 antibody was used to detect the active CASP3 in a western blot assay. The SuperView 488 caspase 3 assay kit (S6007S, US EVERBRIGHT INC, Suzhou, China) was used to detect CASP3 activity in HaEpi cells by immunocytochemistry. The treatment of cells with 10 μM 20E for 24–48 h did not promote cell apoptosis, but the amount of CASP3 green fluorescence significantly increased after the treatment with 10 μM 20E for 72 h, indicating 20E promotes apoptosis in a long-term treatment (S13B Fig). The YF488-Annexin V and propidium iodide (PI) apoptosis kit (Y6002, US EVERBRIGHT INC, Suzhou, China) and flow cytometry were used to detect apoptosis. YF488-Annexin V is Annexin V labeled with the green fluorescent probe YF488, which binds to phosphatidylserine on the cell membrane’s outside to label apoptotic cells. PI is a DNA binding dye that emits red fluorescence in late-stage or necrotic apoptotic cells.

### CCK-8 detected cell viability

CCK-8 assay was used to measure cell viability [67, 68]. CCK-8 cell viability assay kit (C6005M, US EVERBRIGHT INC, Suzhou, China) contains WST-8, which is reduced by dehydrogenase in the mitochondria to a highly water-soluble orange-yellow formazan product in the presence of electron carrier. The depth of the dye is proportional to the number of living cells. After adjusting the cell density to 4 ×10^4^ cells/mL, a 100 μL cell suspension was put to a 96-well plate, followed by 10 μL CCK-8, and incubated for 2–3 h in an incubator. When the color steadied, the absorbance value at 450 nm was determined.

### FFAs determination

Removed 50 μL of hemolymph from larvae and pupae at various developmental stages and mixed with phenylthiourea (PTU) to prevent hemolymph melanization, added reagents as directed (BC0590, Solarbio, Shanghai, China), and measured absorbance at a wavelength of 550 nm by spectrophotometry. The standard curve was created by diluting the standard to various concentrations, and the FFA levels were estimated using the standard curve.

### Glycerol determination

Weighed the fat body at various development stages and rinsed with PBS, added 10 μL of lysis solution per 1 mg of tissue in proportion and ground it quickly on ice (F005-2-1, Njjcbio, Nanjing, China). After incubating samples at 70°C for 10 min to inactivate lipase, they were centrifuged and the supernatant was used to detect at 620 nm using a spectrophotometer. A standard curve was established based on the concentration and absorbance value of the standard substance: y = 0.0002 × x+0.1022, x: glycerol content (μmol/L); y: absorbance. Divided the value by the mass fraction to obtain the glycerol levels (μmol/g).

### FAAs determination

Hemolymph (25 μL) was quickly collected into a 1.5 mL tube containing 1.5 μL 1 mM PTU and 25 μL anticoagulant (450 mM NaCl, 10 mM KCI, 10 mM EDTA, 10 mM HEPES). After centrifuging the samples at 1000 × *g* for 10 min, 30 μL of the supernatant was removed and mixed with 300 μL of 20% trichloroacetic acid. After thorough mixing, the samples were centrifuged at 8000 × *g* for 10 min, the supernatant was removed, filtered through a 0.22 μm filter, and injected into L-8900 Amino Acid Analyzer (Hitachi Limited, Tokyo, Japan) to detect different FAAs levels.

### Hemolymph glucose determination

Hemolymph (30 μL) was collected from at least three insects into tubes containing 1.5 μL of 1 mM PTU and centrifuged at 1000 × *g* for 3 min to remove blood cells. 10 μL of serum was added with 1 mL of glucose extract from glucose determination kit (ml076789, Mlbio, Shanghai, China), incubated at 37°C for 10 min in the dark, and then the absorbance was measured at a wavelength of 505 nm by spectrophotometry. The glucose levels of the samples were calculated according to the formula: Sample tube absorbance/calibration tube absorbance × calibration solution concentration (5.5 mmol/L).

### PA determination

From selected larvae or pupae, the fourth pair of appendages were cut off, 45 μL of hemolymph was collected into a tube containing 2.5 μL 1 mM PTU, added with 200 μL of extract buffer, and quickly ground on ice. After standing for 30 min at room temperature, the samples were centrifuged at 8000 × *g* for 10 min at room temperature. 75 μL supernatant was added with reagent one and two in order, following the instructions of the PA determination kit (BC2205, Solarbio, Shanghai, China). The absorbance was measured at 520 nm using spectrophotometry immediately after the sample was mixed. A standard curve was established based on the concentration and absorbance value of the standard substance: y = 52.439 × x-6.6884; x: absorbance; y: sodium PA levels (μg/mL). The value of sodium PA was obtained from the standard curve and then multiplied by 10 to obtain the levels of PA *in vivo*.

### Trehalose determination

Hemolymph (50 μL) was quickly collected from more than three larvae or pupae from the fourth pair of appendages into a 1.5 mL centrifuge tube containing 2.5 μL 1 mM PTU and centrifuged at 1000 × *g* for 3 min. The supernatant was retained and added with 450 μL of extract in trehalose determination kit (BC0335, Solarbio, Shanghai, China). After standing at room temperature for 45 min, the samples were centrifuged at 8000 × *g* for 10 min. The supernatant (250 μL) were removed and mixed with the working solution in the kit. After heating the samples at 95°C for 10 min and allowing them to cool naturally, the absorbance at 620 nm was measured using spectrophotometry. A standard curve was established based on the absorbance value of the standard: y = 5.6955 × x+0.0908; x: trehalose content (mg/g); y: absorbance. The trehalose value was obtained from the standard curve and multiplied by 10 to calculate the *in vivo* trehalose levels.

### Glycogen determination

The fat body (0.1 g) was extracted at different development stages and rinsed with PBS, added with extract buffer from a glycogen determination kit (ml016886, Mlbio, Shanghai, China), incubated in water bath at 100°C for 20 min, and shaken it every 5 min to fully mix. After the tissue samples were completely cooled, other reagents were added according to the instructions of the kit. The absorbance of each sample was detected at 620 nm using a spectrophotometer, and the glycogen levels were calculated as: Glycogen (mg/g) = tested tube absorbance value/standard tube absorbance value × standard tube content × dilution factor/0.11.

### The antibodies used in this study

The rabbit polyclonal anti-cleaved-CASP3 antibody was provided by WanLei biological company (WL01992, Wanlei, Shenyang, China). Monoclonal anti-ACTB antibody (AC026, ABclonal Technology, Wuhan, China) and monoclonal anti-His antibody (AE003, ABclonal Technology, Wuhan, China) were obtained from ABclonal. The polyclonal rabbit antibodies against *H*. *armigera* LC3 and KLF15 were prepared in our laboratory using recombinantly expressed protein in *E*. *coli* according to previous description [69].

### Bioinformatic analysis

The protein sequences were analyzed using the MEGA 7 software to construct a phylogenetic tree. The DNAMAN software (Lynnon Biosoft, San Ramon, CA, USA) was used to generate the multiple sequence alignment. The reading frames of genes were analyzed at the NCBI (https://www.ncbi.nlm.nih.gov/). The protein structural domains were analyzed using SMART (http://smart.embl-heidelberg.de/). The EcRE motif was predicted by JASPAR transcription factor database (http://jaspar.genereg.net/).

### Statistical analysis

We used Excel and GraphPad 7 software (GraphPad Inc., La Jolla, CA, USA) to analyze the data and generate the figures. All metabolites were measured from at least three larvae or pupae, and the experiments were repeated three times. The paired data were analyzed statistically using Student’s t-test, asterisks represent significant differences (* *p* < 0.05, ** *p* < 0.01, *** *p* < 0.001). In the figures, the bars represent the mean ± standard deviation (SD) for three independent biological experiments. The comparison between multiple sets of data was analysis of variance (ANOVA). The different lowercase letters showed significant differences (*p* < 0.05).

## Supporting information

S1 Fig A. Phylogenetic tree analysis of KLFs from different species. There are five *Klfs* in the genome, *Luna*, LOC110376548 (XP_021190718.1, XP_021190719.1, XP_021190721.1); *Dar*, LOC110374594 (XP_021188035.1, XP_021188036.1, XP_021188037.1); *Klf8*, LOC110378525 (XP_021193494.1); *Klf9*, LOC110384292 (XP_021201196.1); *Klf16*, LOC110373997 (XP_021187184.1). *H*. *armigera*: *Helicoverpa armigera*; *B*. *mori*: *Bombyx mori*; *H*. *sapiens*: *Homo sapiens*; *D*. *melanogaster*: *Drosophila melanogaster*; *C*. *elegans*: *Caenorhabditis elegans*.
(TIF)

S2 Fig **A.** Phylogenetic tree analysis of KLFs from *H*. *armigera and D*. *melanogaster*. **B.** Sequence alignment of *H*. *sapiens* KLF9 and KLF15, *D*. *melanogaster* KLF15, and *H*. *armigera* KLF15 to name the targeted gene correctly. The area marked in the red box is the structural domain.
(TIF)

S3 Fig Screened *Klf* that highly expressed in the fat body and involved in gluconeogenesis. **A-E.** Reverse transcription polymerase chain reaction (RT-PCR) screened highly expressed *Klfs* in the fat body. E: epidermis; M: midgut; F: fat body. **F.** The RNAi efficiency of *Luna*, *Klf15* and *Klf16* in the fat body. **G.** Glucose levels were detected in the hemolymph after knockdown *Luna*, *Klf15* and *Klf16*. **H.** Detected the expression of *G6p* and *Pepck* after *Klf15*, *Klf16* knockdown by qRT-PCR.
(TIF)

S4 Fig Screened EcRE bs on the *Klf15* promoter. **A.** Alignment of the EcRE sites in the promoter of *Klf15* predicted by JASPAR transcription factor database. **B.** Genomic amplification of sequences containing EcRE in the *Klf15* promoter and incubated with EcR nuclear proteins for EMSA to screen the EcRE site that bind to EcR.
(TIF)

S5 Fig **A.** qRT-PCR validation of the interference efficiency of *Klf15* in HaEpi cells. **B.** *GFP* expression was detected after *YFP* was knocked down.
(TIF)

S6 Fig **A.** Flow cytometry analysis of YF488-annexin V and PI staining after knocked down *YFP* and *Klf15*. R1, normal cells; R2, early apoptotic cells; R3, middle and late apoptotic cells; R4, necrotic cells. **Ai.** Statistical analysis for A. **B.** CCK-8 detected cell viability after knocked down *YFP* and *Klf15*.
(TIF)

S7 Fig Detected the expression of all KLF bs-containing *Atgs* after first injection dsRNA for 72 h. (TIF)

S8 Fig **A.** The RNAi efficiency of *Atg8* and *Casp3* in the epidermis, midgut, and fat body assessed using qRT-PCR after the third injection of dsRNA. **B.** HE staining, Nile red staining and TUNEL staining showing the morphology of fat body after *dsCasp3* injection. **C.** Glucose levels in the hemolymph decreased after knockdown *Casp3*.
(TIF)

S9 Fig Trehalose levels in the hemolymph after first injection dsRNA for 72 h. (TIF)

S10 Fig **A.** RFP-His, KLF15-RFP-His detection by western blotting. **B.** RFP-His and KLF15-RFP-His fluorescent plasmid transfection efficiency detection. **C.** qRT-PCR showing the expression of *Atg8*, *Casp3*, *G6p* and *Pepck* after overexpression of RFP-His and KLF15-RFP-His in HaEpi cells.
(TIF)

S11 Fig Antigen expression of KLF15 by SDS-PAGE and specific detection. **A.** 564 bp length KLF15 was overexpressed in *E*. *coli* via the pET-30a plasmid. The expression product was 32 kDa (20 kDa KLF15 plus 12 kDa his-tag). Lane 1, protein lysates of *E*. *coli* transformed with plasmid pET-30a-KLF15 before IPTG induction. Lane 2, protein lysates after IPTG (0.5 mM) induction overnight at 37°C. Lane 3, the supernatant of *E*. *coli* lysates. Lane 4, the precipitate of *E*. *coli* lysates. Lane 5, the purified KLF15 used for rabbit antibody preparation. **B.** Specificity detection of KLF15 antibody with epidermis of the 6th-96 h larvae. **C-E.** Specificity detection of β-Actin, LC3 and cleaved-CASP3 antibodies with fat body of the 6th-96 h larvae.
(TIF)

S12 Fig TEM observation after injection with dsRNA in the fat body under large field view. The area pointed by the red arrows includes autophagosomes contained degenerating cytoplasmic organelles or degraded lipid droplets, and autophagic vacuoles, the blue arrows represents the apoptotic nuclei. The bars represent 100 μm.
(TIF)

S13 Fig 20E promoted autophagy and apoptosis. **A.** Transfection of RFP-GFP-LC3-His fluorescent double-labeled plasmid in HaEpi cells. 5 μM 20E to stimulate 1 h, 12 h, 24 h and 48 h, respectively, DMSO was used as the control. The yellow bars represented 20 μm. The yellow arrows represented autophagosome puncta, the blue arrow represented autolysosome puncta. **B.** Examination of apoptosis in HaEpi cells by the SuperView 488 caspase 3 assay kit. 10 μM 20E to stimulate 24 h, 48 h and 72 h, respectively. The yellow bars represented 100 μm.
(TIF)

S1 Table PCR primer sequences and GenBank accession numbers of genes used in the experiments. (DOCX)
