# Supplementary figures and images for: Krüppel-like factor 15 integrated autophagy and gluconeogenesis to maintain glucose homeostasis under 20-hydroxyecdysone regulation
Source: PLoS Genet. 2022 Jun 13;18(6):e1010229. doi: 10.1371/journal.pgen.1010229 (PMC9191741; doi:10.1371/journal.pgen.1010229)

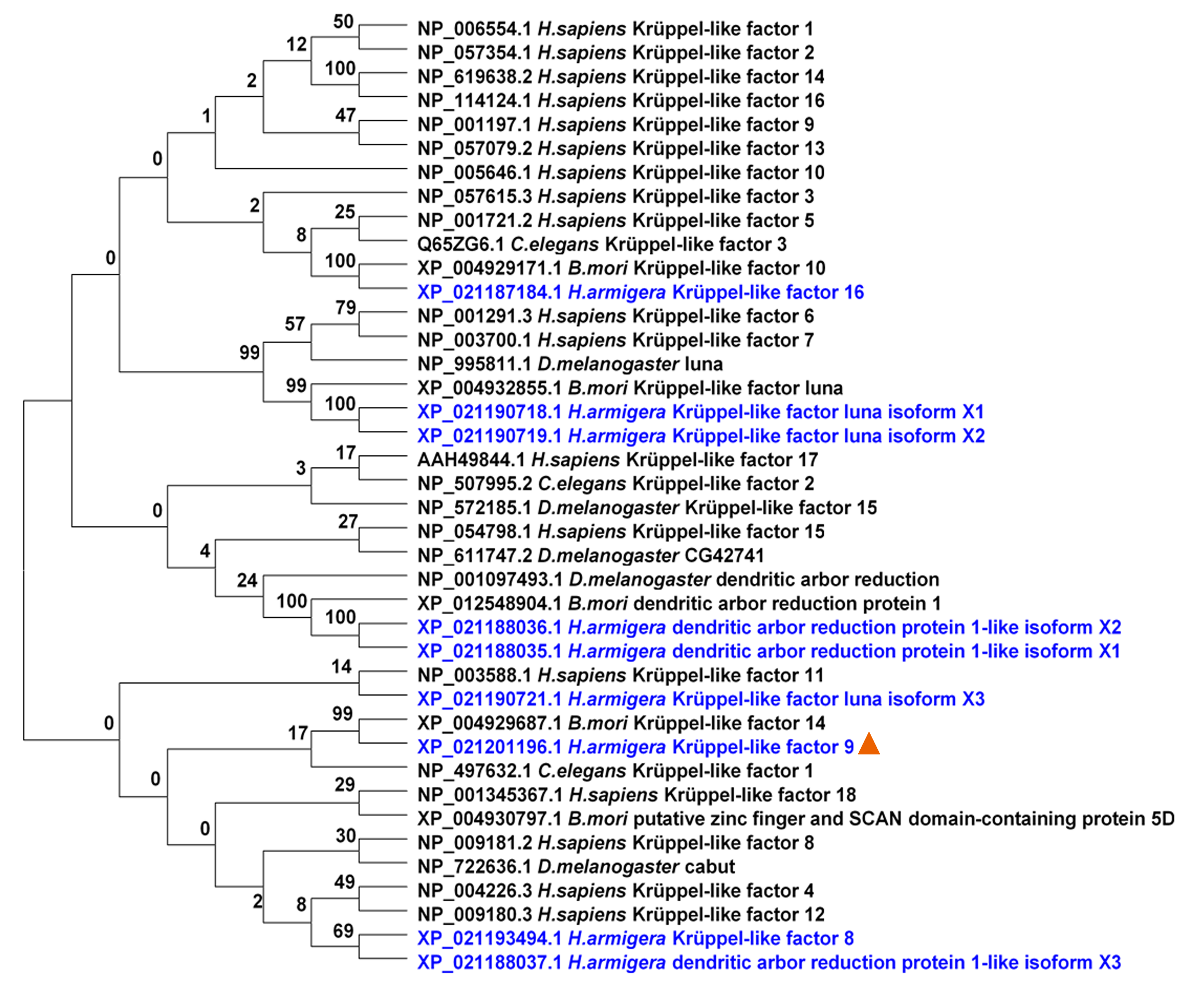

Supplement: S1 Fig — There are five Klfs in the genome, Luna, LOC110376548 (XP_021190718.1, XP_021190719.1, XP_021190721.1); Dar, LOC110374594 (XP_021188035.1, XP_021188036.1, XP_021188037.1); Klf8, LOC110378525 (XP_021193494.1); Klf9, LOC110384292 (XP_021201196.1); Klf16, LOC110373997 (XP_021187184.1). H. armigera: Helicoverpa armigera; B. mori: Bombyx mori; H. sapiens: Homo sapiens; D. melanogaster: Drosophila melanogaster; C. elegans: Caenorhabditis elegans. (TIF) [file pgen.1010229.s001.tif]

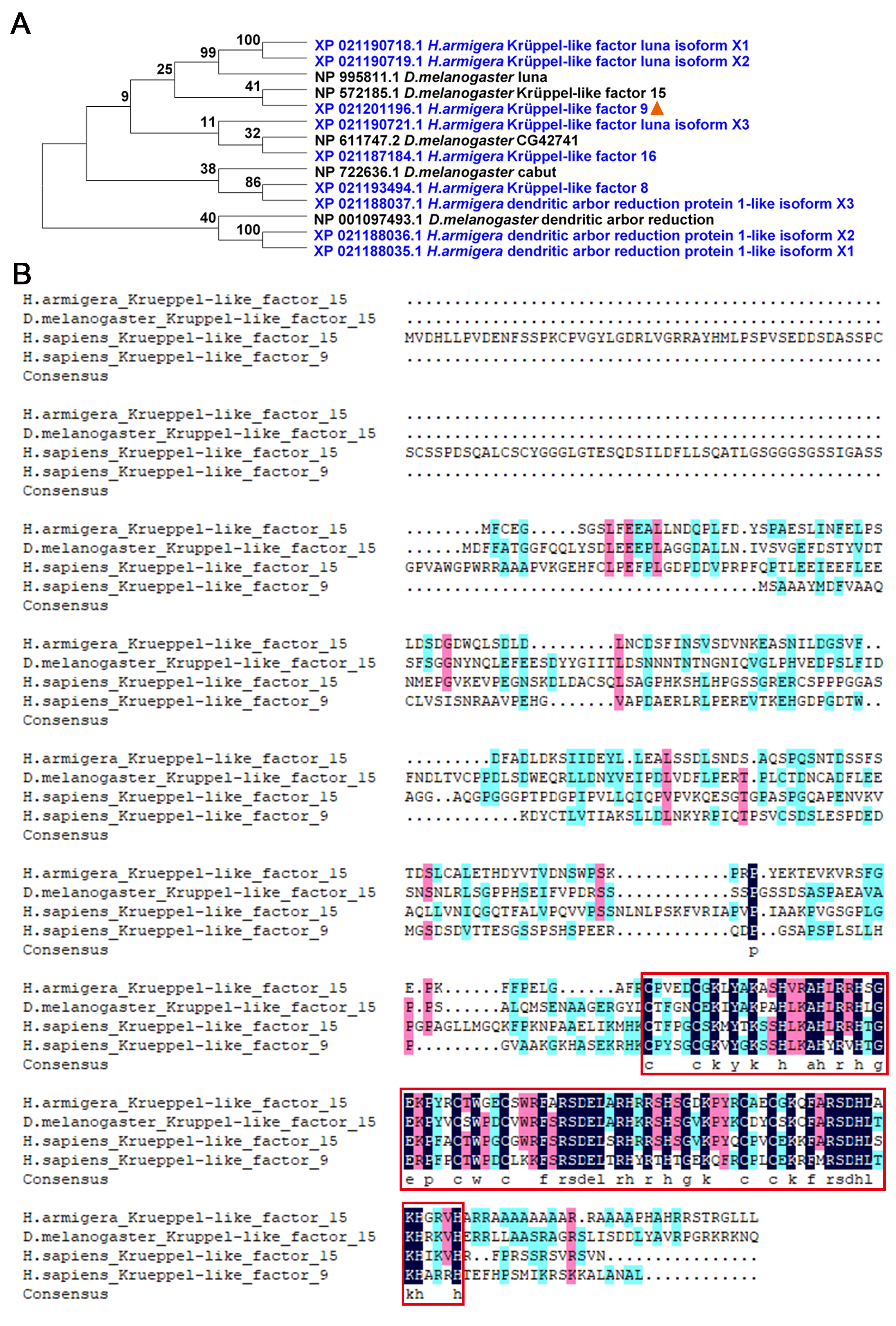

Supplement: S2 Fig — A. Phylogenetic tree analysis of KLFs from H. armigera and D. melanogaster. B. Sequence alignment of H. sapiens KLF9 and KLF15, D. melanogaster KLF15, and H. armigera KLF15 to name the targeted gene correctly. The area marked in the red box is the structural domain. (TIF) [file pgen.1010229.s002.tif]

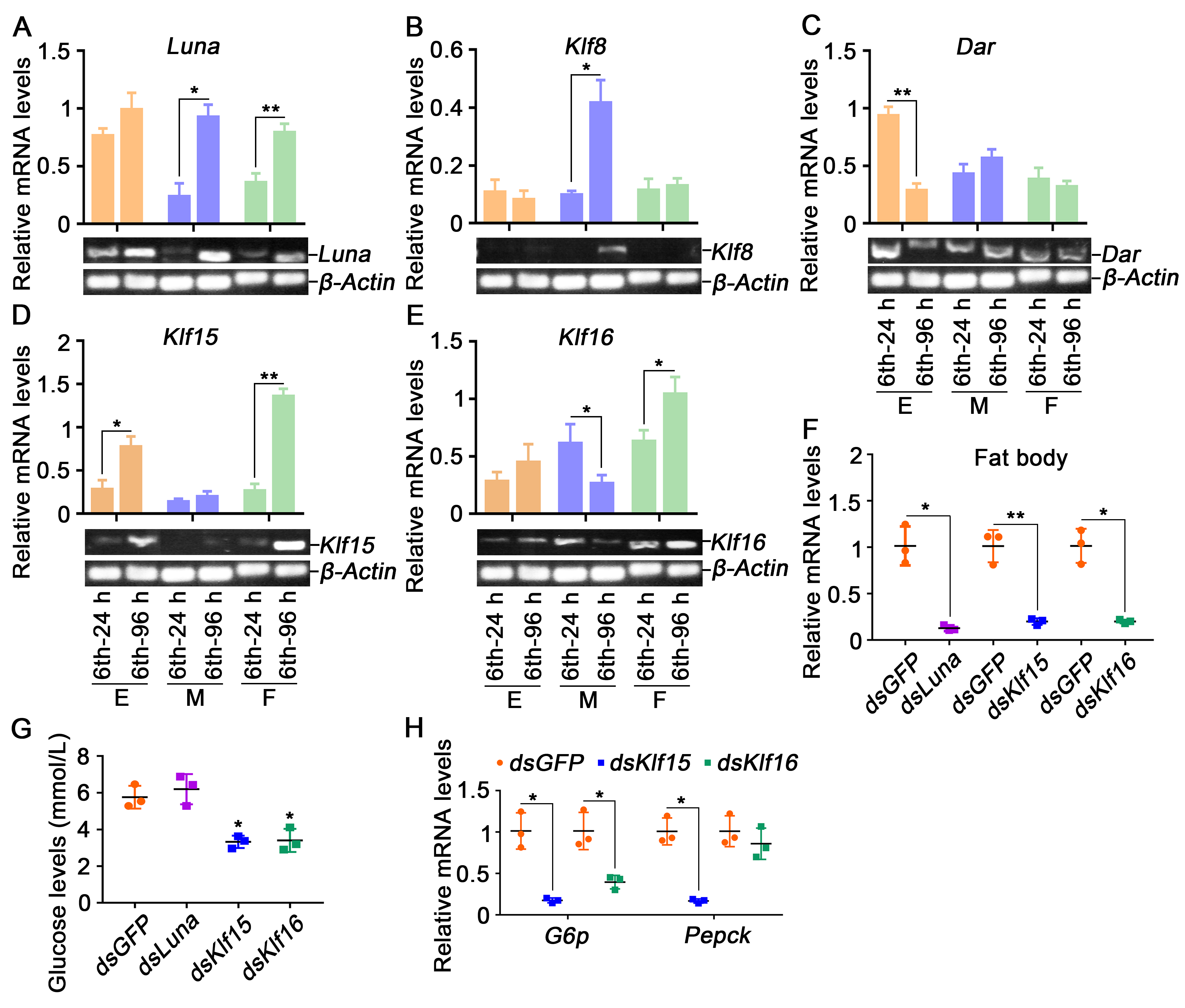

Supplement: S3 Fig — A-E. Reverse transcription polymerase chain reaction (RT-PCR) screened highly expressed Klfs in the fat body. E: epidermis; M: midgut; F: fat body. F. The RNAi efficiency of Luna, Klf15 and Klf16 in the fat body. G. Glucose levels were detected in the hemolymph after knockdown Luna, Klf15 and Klf16. H. Detected the expression of G6p and Pepck after Klf15, Klf16 knockdown by qRT-PCR. (TIF) [file pgen.1010229.s003.tif]

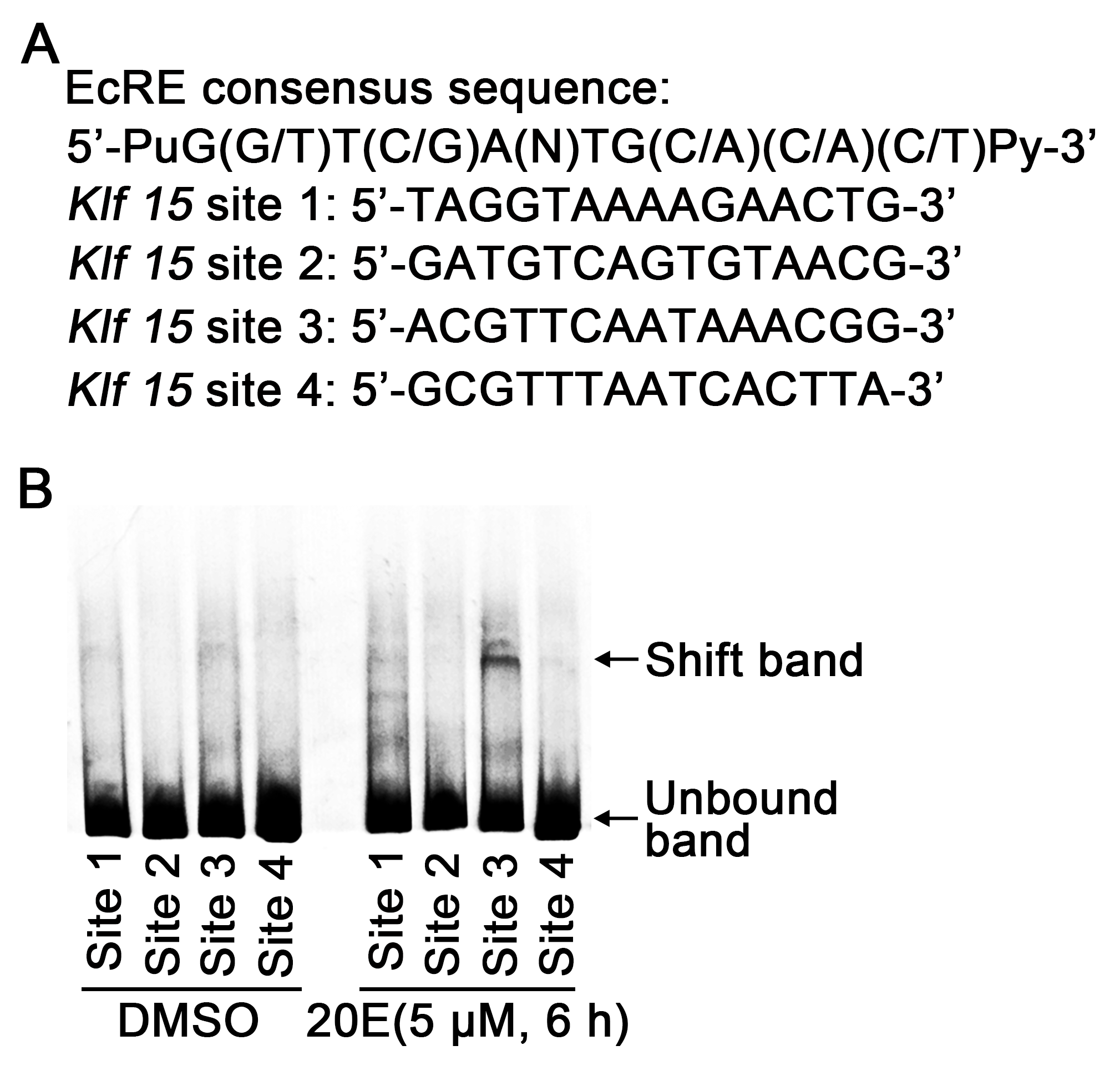

Supplement: S4 Fig — A. Alignment of the EcRE sites in the promoter of Klf15 predicted by JASPAR transcription factor database. B. Genomic amplification of sequences containing EcRE in the Klf15 promoter and incubated with EcR nuclear proteins for EMSA to screen the EcRE site that bind to EcR. (TIF) [file pgen.1010229.s004.tif]

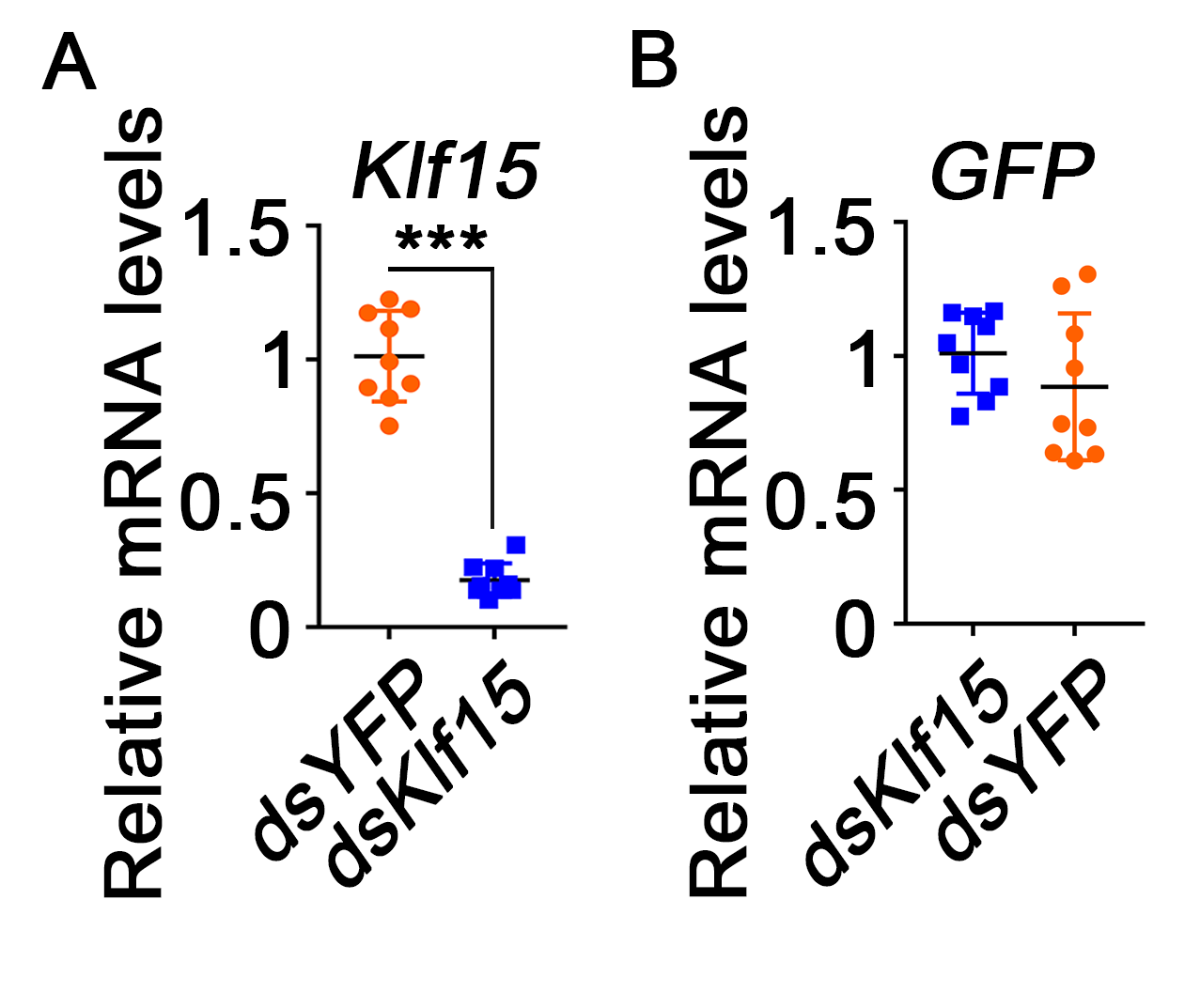

Supplement: S5 Fig — A. qRT-PCR validation of the interference efficiency of Klf15 in HaEpi cells. B. GFP expression was detected after YFP was knocked down. (TIF) [file pgen.1010229.s005.tif]

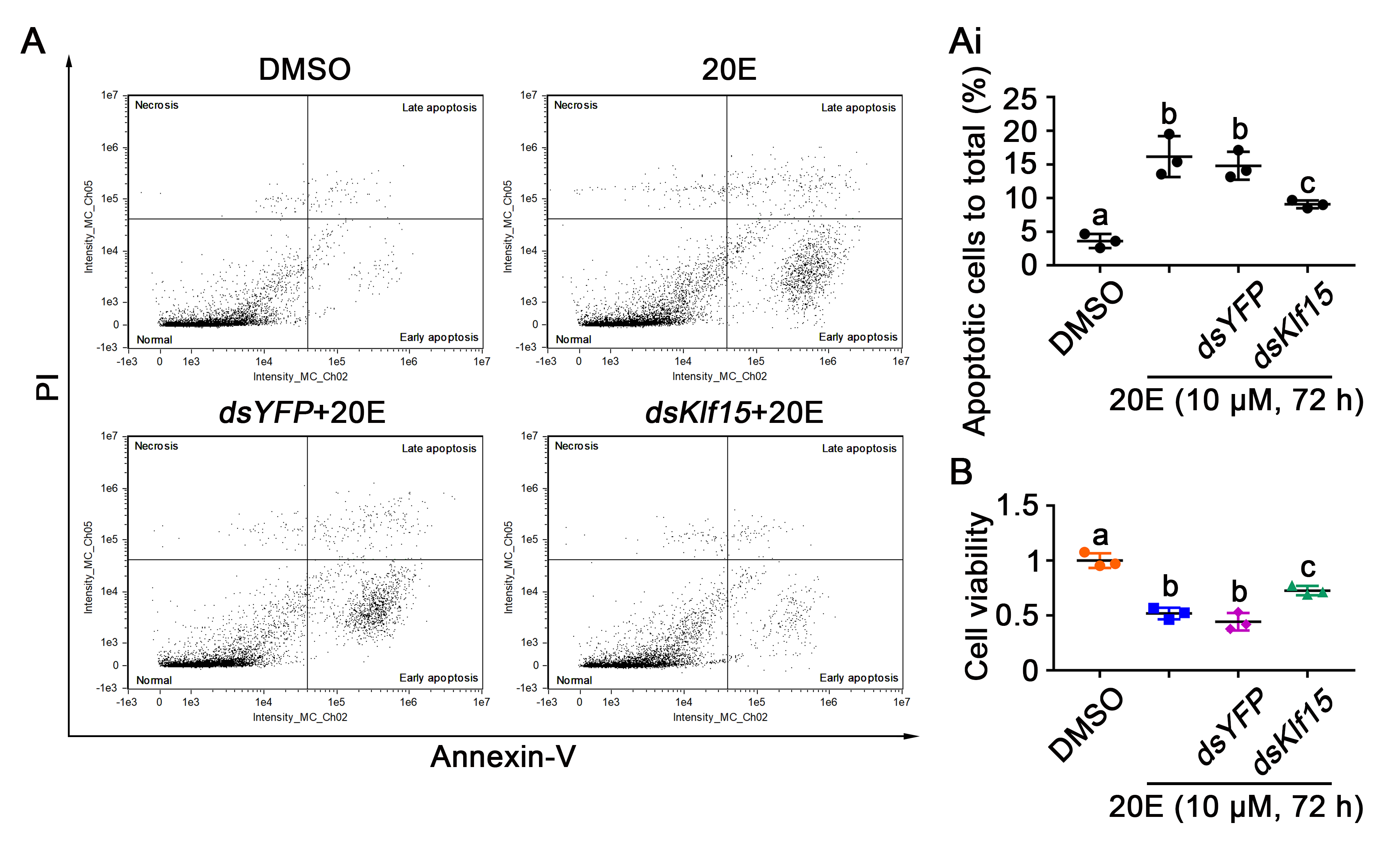

Supplement: S6 Fig — A. Flow cytometry analysis of YF488-annexin V and PI staining after knocked down YFP and Klf15. R1, normal cells; R2, early apoptotic cells; R3, middle and late apoptotic cells; R4, necrotic cells. Ai. Statistical analysis for A. B. CCK-8 detected cell viability after knocked down YFP and Klf15. (TIF) [file pgen.1010229.s006.tif]

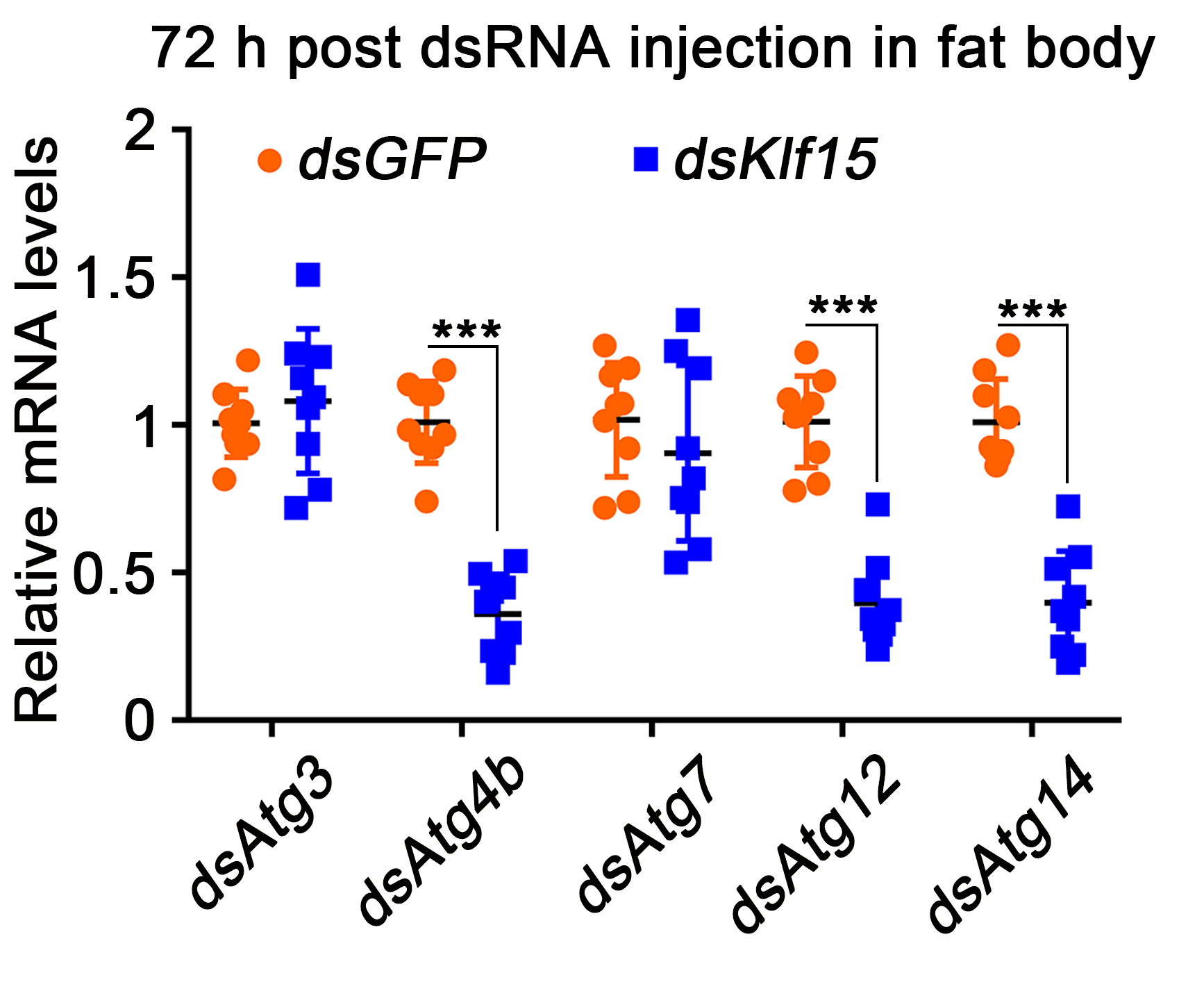

Supplement: S7 Fig — (TIF) [file pgen.1010229.s007.tif]

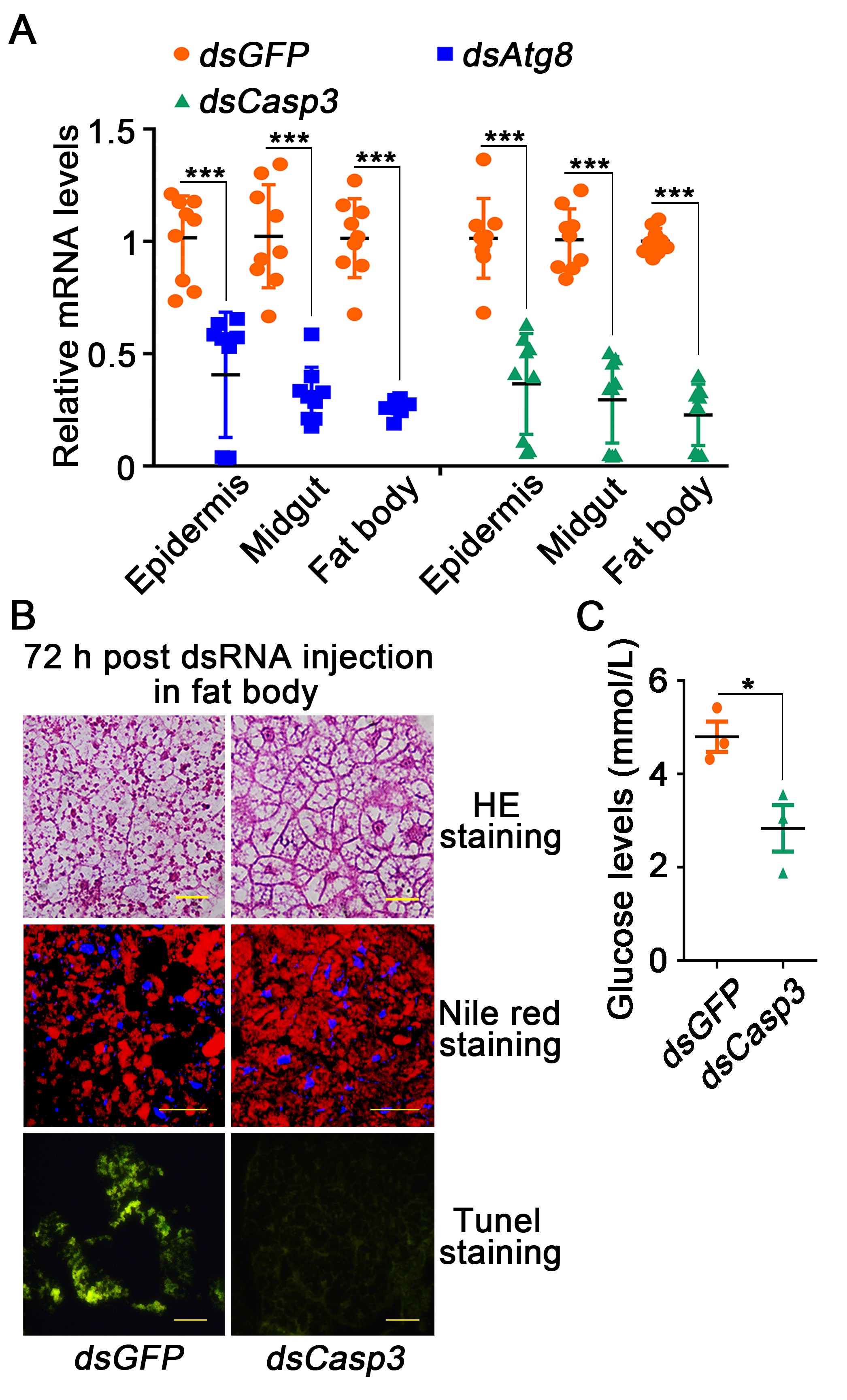

Supplement: S8 Fig — A. The RNAi efficiency of Atg8 and Casp3 in the epidermis, midgut, and fat body assessed using qRT-PCR after the third injection of dsRNA. B. HE staining, Nile red staining and TUNEL staining showing the morphology of fat body after dsCasp3 injection. C. Glucose levels in the hemolymph decreased after knockdown Casp3. (TIF) [file pgen.1010229.s008.tif]

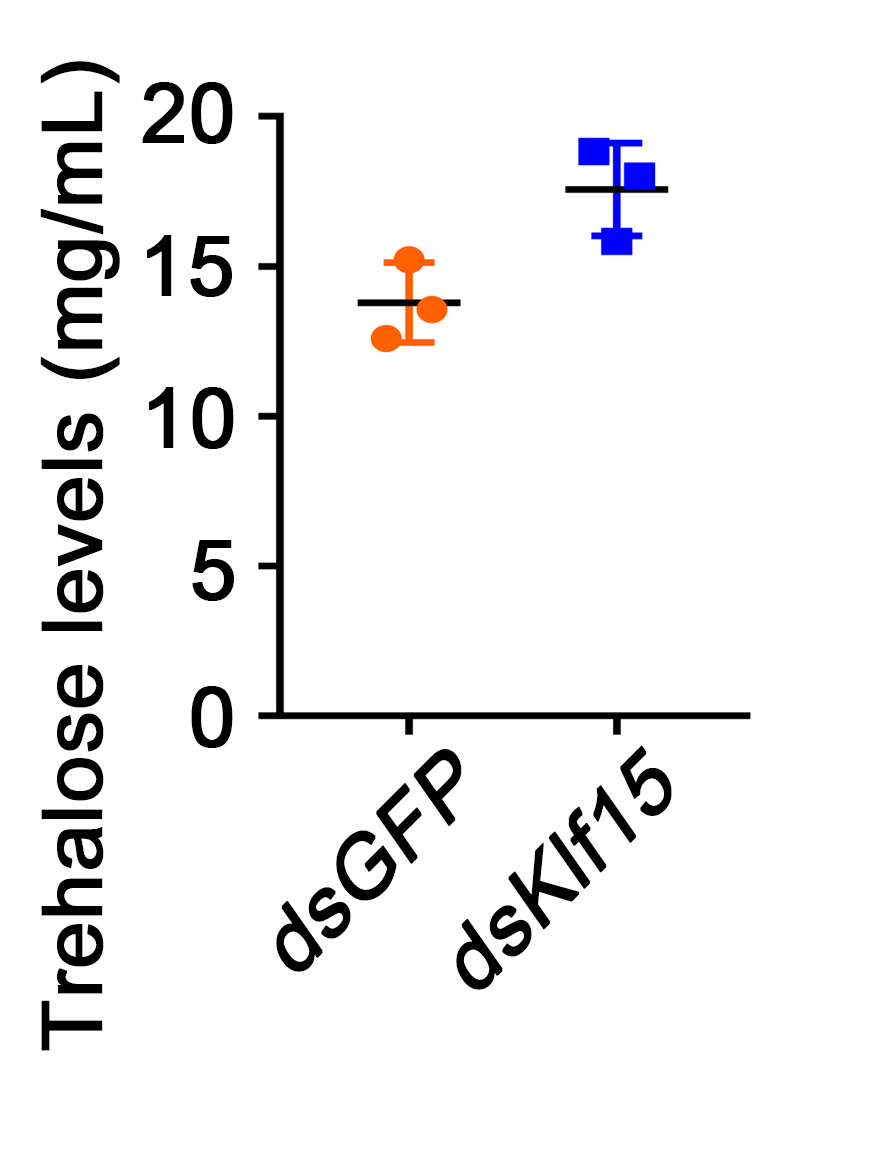

Supplement: S9 Fig — (TIF) [file pgen.1010229.s009.tif]

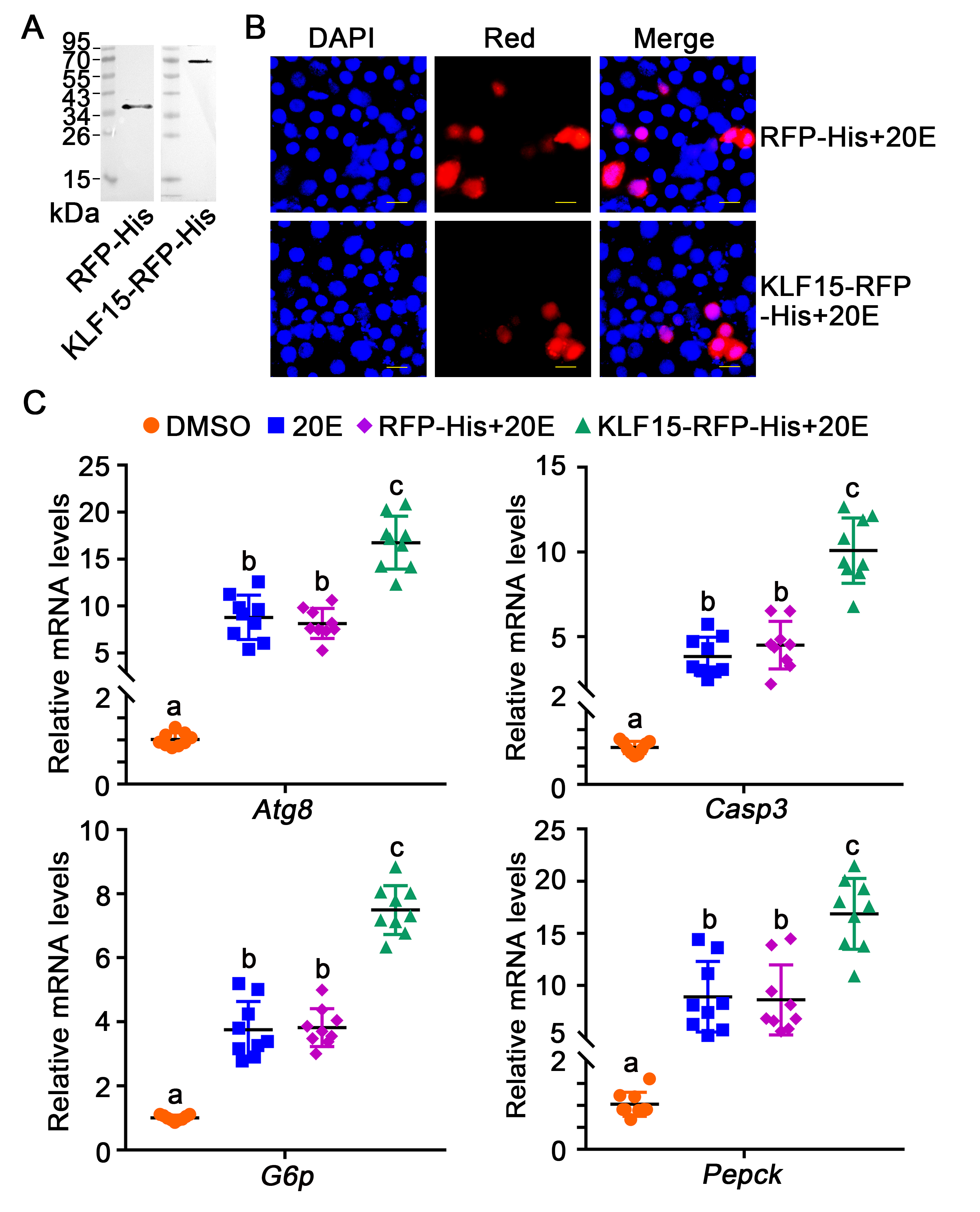

Supplement: S10 Fig — A. RFP-His, KLF15-RFP-His detection by western blotting. B. RFP-His and KLF15-RFP-His fluorescent plasmid transfection efficiency detection. C. qRT-PCR showing the expression of Atg8, Casp3, G6p and Pepck after overexpression of RFP-His and KLF15-RFP-His in HaEpi cells. (TIF) [file pgen.1010229.s010.tif]

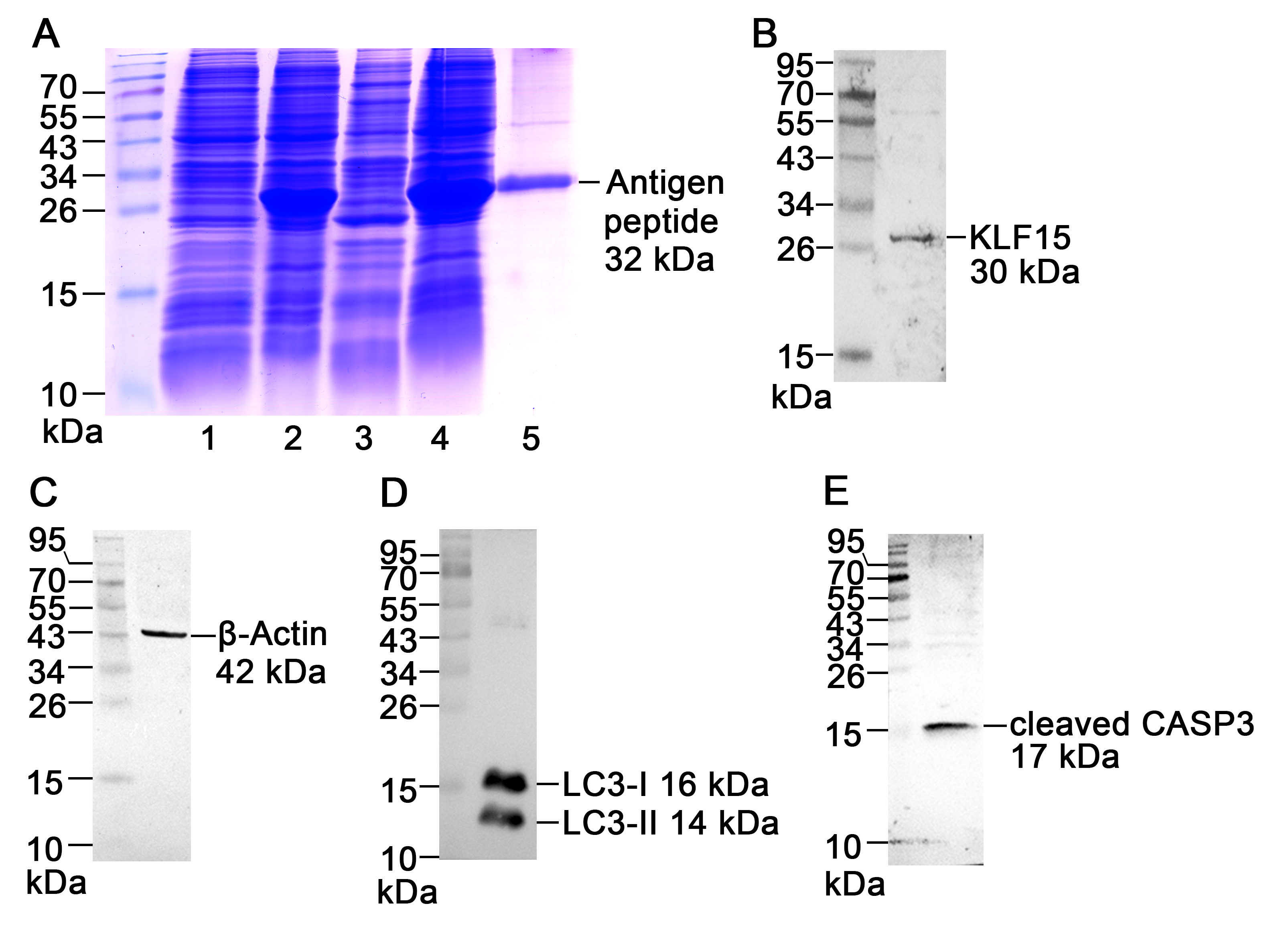

Supplement: S11 Fig — A. 564 bp length KLF15 was overexpressed in E. coli via the pET-30a plasmid. The expression product was 32 kDa (20 kDa KLF15 plus 12 kDa his-tag). Lane 1, protein lysates of E. coli transformed with plasmid pET-30a-KLF15 before IPTG induction. Lane 2, protein lysates after IPTG (0.5 mM) induction overnight at 37°C. Lane 3, the supernatant of E. coli lysates. Lane 4, the precipitate of E. coli lysates. Lane 5, the purified KLF15 used for rabbit antibody preparation. B. Specificity detection of KLF15 antibody with epidermis of the 6th-96 h larvae. C-E. Specificity detection of β-Actin, LC3 and cleaved-CASP3 antibodies with fat body of the 6th-96 h larvae. (TIF) [file pgen.1010229.s011.tif]

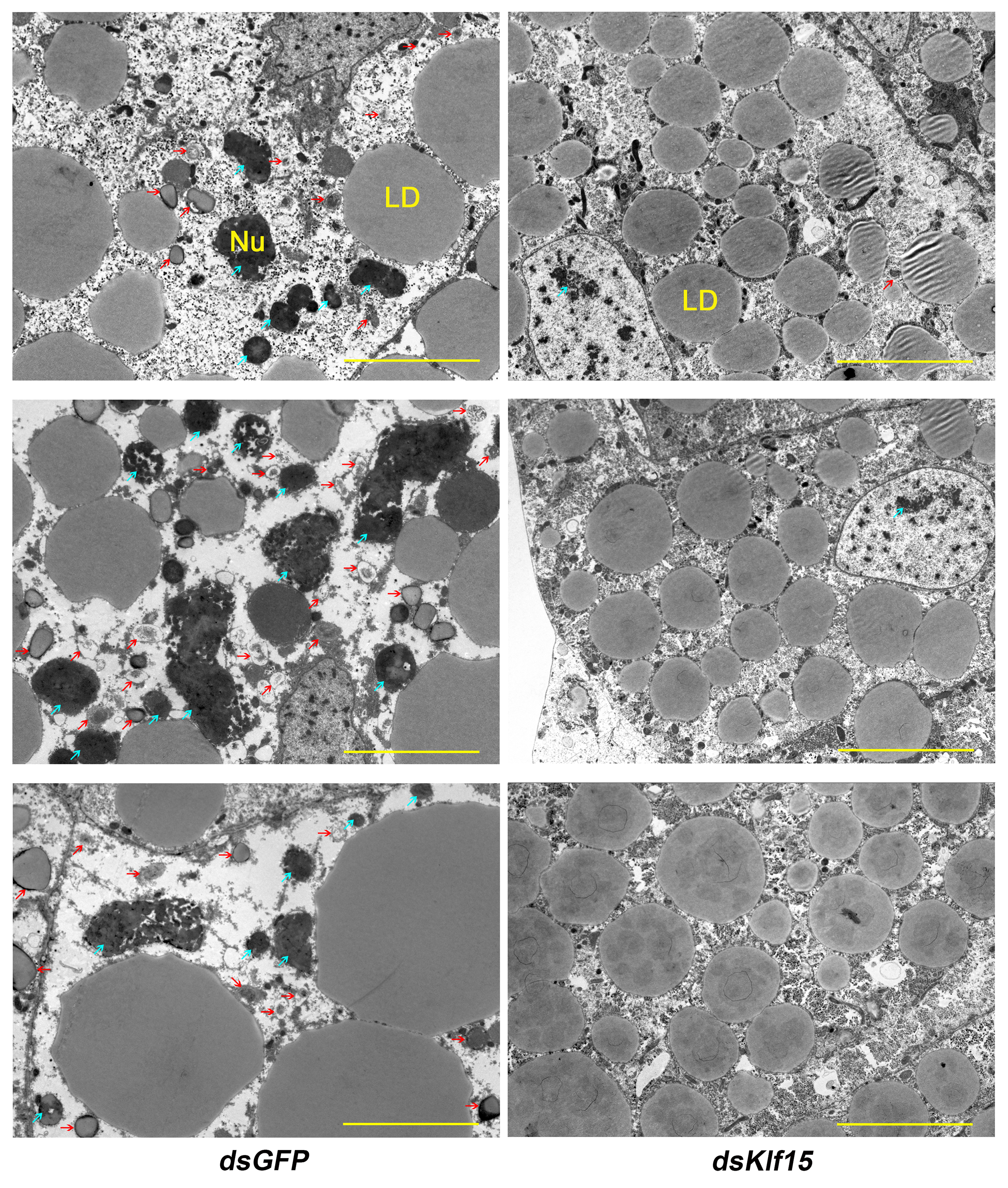

Supplement: S12 Fig — The area pointed by the red arrows includes autophagosomes contained degenerating cytoplasmic organelles or degraded lipid droplets, and autophagic vacuoles, the blue arrows represents the apoptotic nuclei. The bars represent 100 μm. (TIF) [file pgen.1010229.s012.tif]

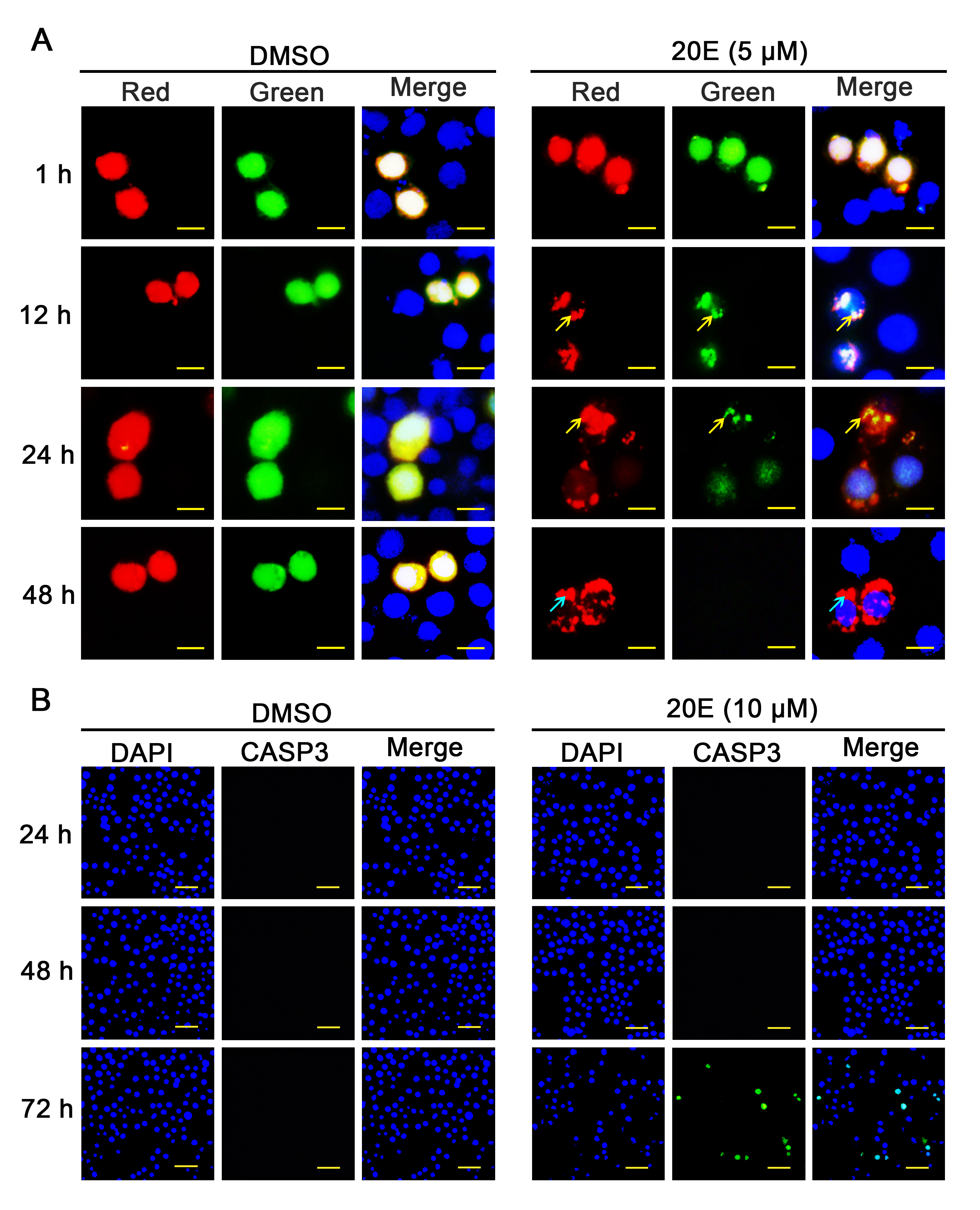

Supplement: S13 Fig — A. Transfection of RFP-GFP-LC3-His fluorescent double-labeled plasmid in HaEpi cells. 5 μM 20E to stimulate 1 h, 12 h, 24 h and 48 h, respectively, DMSO was used as the control. The yellow bars represented 20 μm. The yellow arrows represented autophagosome puncta, the blue arrow represented autolysosome puncta. B. Examination of apoptosis in HaEpi cells by the SuperView 488 caspase 3 assay kit. 10 μM 20E to stimulate 24 h, 48 h and 72 h, respectively. The yellow bars represented 100 μm. (TIF) [file pgen.1010229.s013.tif]
