# Supplementary material for: Krüppel-like factor 15 integrated autophagy and gluconeogenesis to maintain glucose homeostasis under 20-hydroxyecdysone regulation
Source: PLoS Genet. 2022 Jun 13;18(6):e1010229. doi: 10.1371/journal.pgen.1010229 (PMC9191741; doi:10.1371/journal.pgen.1010229)
Supplement: S1 Table — (DOCX) [file pgen.1010229.s014.docx]

**S1 Table. PCR primer sequences and GenBank accession numbers of genes used in the experiments.**

| **Primer name** | | | **Sequence (5’-3’)** | | | **GenBank accession number** |  |
| --- | --- | --- | --- | --- | --- | --- | --- |
| **qRT-PCR** | | |  | | |  |  |
| *Tre*-RTF | | | aatggcgaggctctacaat | | | AJK29979.1 |  |
| *Tre*-RTR | | | atggctccagttcgttgtc | | |  |  |
| *Tps*-RTF  *Tps*-RTR | | | atgctgagcccaaactcttcg  ctttcccttctgttgtttgagc | | | XP_021201246 |  |
| *Gp*-RTF  *Gp*-RTR | | | gagatggcctaccacgat  gaaccaacaacgcacaag | | | XP_021190210.1 |  |
| *Gs*-RTF | | | tcggacttattatgctcaggac | | | XP_021199514.1 |  |
| *Gs*-RTR | | | aggagatgttctgcctcgtaa | | |  |  |
| *G6p*-RTF  *G6p*-RTR | | | gatggaacaaatctacgcactcg  agccaccatttcataagagcatt | | | XP_021186784.1 |  |
| *Pepck*-RTF  *Pepck*-RTR | | | cgcaagaacgatgagggcaaat  gttgtcacagcggcgcaggat | | | XP_021197900.1 |  |
| *Atg8*-RTF | | | aagagaaagaccgaaggcg | | | XP_021181573.1 |  |
| *Atg8*-RTR | | | ggtctccgagtctagccttc | | |  |  |
| *Casp3*-RTF | | | acccttcaaatacgagcaatcc | | | AEK20819.1 |  |
| *Casp3*-RTR | | | catcattgtccgtaccattcctt | | |  |  |
| *Hk*-RTF  *Hk*-RTR | | tcacattctcgttcccgatgaa  gtgccggtggtgtcgttga | | XM_021342737.1 | | |  |
| *Pfk*-RTF | | | ctgatgtgactgggtgggtag | | | ALJ94066.1 |  |
| *Pfk*-RTR | | | cgggcacgttgttactgat | | |  |  |
| *Pk*-RTF | | | agggtgggcaaaccagtaatc | | | ARQ20739.1 |  |
| *Pk*-RTR | | | tcggacctcaacaaccaaatca | | |  |  |
| *Klf15*-RTF | tgttttgtgaaggctctggat | | | | XM_021345521.1 | | |
| *Klf15*-RTR | | | gatctaaatcactaagctgcc | | |  |  |
| *Klf16*-RTF | | | tcgtcccacctgaaagcg | | | XP_021187184.1 |  |
| *Klf16*-RTR | | | cggcacccgaacttcttc | | |  |  |
| *Klf8*-RTF | | | cgtgctgccctctacaat | | | XP_021193494.1 |  |
| *Klf8*-RTR | | | gacaaccagccacatcac | | |  |  |
| *Dar*-RTF  *Dar*-RTR | | | agcaccagccaaacacctcctt  gcagtggtggaccctcctcttct | | | XP_021188037.1 |  |
| *Luna*-RTF  *Luna*-RTR | | | caacccaccacgaccttca  cgccagtatgcgtcctcttat | | | XP_021190721.1 |  |
| *EcR*-RTF | | | aattgcccgtcagtacga | | | ACD74807.1 |  |
| *EcR*-RTR | | | tgagcttctcattgagga | | |  |  |
| *β-actin*-RTF | | | cctggtattgctgaccgtatgc | | | EU52707 |  |
| *β-actin*-RTR  *Atg3*-RTF  *Atg3*-RTR  *Atg4b*-RTF  *Atg4b*-RTR  *Atg7*-RTF  *Atg7*-RTR  *Atg12*-RTF  *Atg12*-RTR  *Atg14*-RTF  *Atg14*-RTR | | | ctgttggaaggtggagagggaa  ctggcttgttggatgaggtg  cggcgtttggtagtatttgt  cagaaggcaaggaagtgggc  tgagcggcactatcaacagc  gcgatgacggatgaaacag  agcagccgagatcaacacc  ctacaggcaatgctccaa  ctggtgacggtgcaaacg  gcggaactcaggagccataa  cctccgtgtcctcgcctat | | | XM_021341398.1  XM_021327178.1  XM_021331200.1  XM_021338187.1  XM_021335677.1 |  |
| **RNAi** | | |  | | |  |  |
| *EcR*-RNAiF | | | gcgtaatacgactcactataggcgctggtataacaacggagga | | |  |  |
| *EcR*-RNAiR | | | gcgtaatacgactcactataggaagctggagacaactcctcacg | | |  |  |
| *GFP*-RNAiF | | | gcgtaatacgactcactataggtggtcccaattctcgtggaac | | |  |  |
| *GFP*-RNAiR | | | gcgtaatacgactcactataggcttgaagttgaccttgatgcc | | |  |  |
| *Klf15*-RNAiF | | | gcgtaatacgactcactataggtctcctgcggaaagtcttata | | |  |  |
| *Klf15*-RNAiR | | | gcgtaatacgactcactataggcctctactgggcaacgaaaa | | |  |  |
| *Atg8*-RNAiF | | | gcgtaatacgactcactataggtatcctgatcgtgttccggtaat | | |  |  |
| *Atg8*-RNAiR | | | gcgtaatacgactcactataggttaatatccatatacattctc | | |  |  |
| *Casp3*-RNAiF | | | gcgtaatacgactcactataggacctcgtgcatgtgtgtca | | |  |  |
| *Casp3*-RNAiR  *YFP*-RNAiF  *YFP*-RNAiR  Chip-*Atg8*F  Chip-*Atg8*R  Chip-*Pepck*F  Chip-*Pepck*R  Chip-*Klf15*F  Chip-*Klf15*R | | | gcgtaatacgactcactataggtggaaggcgtgtatgtggt  gcgtaatacgactcactataggcgccagcacgacttcttca  gcgtaatacgactcactataggcgaactccagcaggaccatg  tcgattgttcattctatgactat  tcgctccctatttcgtcaatttc  ttttattcatttggcaccc  acaagatggcgtcagggtg  gcaaagagttacatagtac  cagtagaaaatatcaatta | | |  |  |
| **Exp in *E. Coli*** | | |  | | |  |  |
| Anti-*Klf15*F | | | tactcaggatccatgttttgtgaaggctctggat | | |  |  |
| Anti-*Klf15*R | | | tactcagagctcaccactgtgccgacgtaagtg | | |  |  |
